# Clinically approved immunomodulators ameliorate behavioral changes in a mouse model of hereditary spastic paraplegia type 11

**DOI:** 10.3389/fnins.2024.1299554

**Published:** 2024-02-16

**Authors:** Michaela Hörner, Sandy Popp, Julien Branchu, Giovanni Stevanin, Frédéric Darios, Stephan Klebe, Janos Groh, Rudolf Martini

**Affiliations:** ^1^Section of Developmental Neurobiology, Department of Neurology, University Hospital Würzburg, Würzburg, Germany; ^2^Division of Neurodegenerative Diseases, Department of Neurology, Heidelberg University Hospital and Faculty of Medicine, Heidelberg, Germany; ^3^Center of Mental Health, Department of Psychiatry, Psychosomatics and Psychotherapy, University of Würzburg, Würzburg, Germany; ^4^TSE Systems GmbH, Berlin, Germany; ^5^Institut du Cerveau – Paris Brain Institute, Inserm, Sorbonne Université, Paris, France; ^6^EVerZom, Paris, France; ^7^INCIA, CNRS, EPHE, Université de Bordeaux, Bordeaux, France; ^8^Department of Neurology, University Hospital Essen, Essen, Germany; ^9^Institute of Neuronal Cell Biology, Technical University Munich, Munich, Germany

**Keywords:** adaptive immune system, neuroinflammation, behavioral abnormalities, repurposing drugs, social behavior

## Abstract

We have previously demonstrated that neuroinflammation by the adaptive immune system acts as a robust and targetable disease amplifier in a mouse model of Spastic Paraplegia, type 11 (SPG11), a complicated form of Hereditary Spastic Paraplegia (HSP). While we identified an impact of neuroinflammation on distinct neuropathological changes and gait performance, neuropsychological features, typical and clinically highly relevant symptoms of complicated HSPs, were not addressed. Here we show that the corresponding SPG11 mouse model shows distinct behavioral abnormalities, particularly related to social behavior thus partially reflecting the neuropsychological changes in patients. We provide evidence that some behavioral abnormalities can be mitigated by genetic inactivation of the adaptive immune system. Translating this into a clinically applicable approach, we show that treatment with the established immunomodulators fingolimod or teriflunomide significantly attenuates distinct behavioral abnormalities, with the most striking effect on social behavior. This study links neuroinflammation to behavioral abnormalities in a mouse model of SPG11 and may thus pave the way for using immunomodulators as a treatment approach for SPG11 and possibly other complicated forms of HSP with neuropsychological involvement.

## Introduction

Genetically mediated disorders of the central nervous system (CNS) are usually orphan diseases with poor treatment options (Groh and Martini, 2017). Lack of knowledge of the basic pathomechanism is often the cause of limited therapeutic options. The situation is even more complicated since many genetically mediated diseases are characterized by not only physical manifestations, like motor symptoms, but display additional psychological involvement. Neuropsychological features are often present in many CNS diseases like Alzheimer’s disease (AD), Parkinson’s disease (PD), Multiple Sclerosis (MS), and multiple system atrophy (Becker et al., 1988; St Clair et al., 1988; Brassington and Marsh, 1998; Barcelos et al., 2018). However, despite its substantial relevance for patients, relatives, and treating clinicians, the pathomechanisms leading to neuropsychological symptoms remain mainly elusive. Interestingly, primary neuropsychological disorders are often associated with low-grade secondary inflammation, as seen for example in depressive disorder (Miller and Raison, 2016; Beurel et al., 2020; Majd et al., 2020), schizophrenia (Kirkpatrick and Miller, 2013; Upthegrove and Khandaker, 2020; Sun et al., 2022), bipolar disorder (Pereira et al., 2021; Saccaro et al., 2021), and borderline personality disorder (MacDowell et al., 2020; Saccaro et al., 2021). In a mouse mutant related to abnormal CNS myelin, displaying catatonic features, secondary inflammation has also been shown to lead to behavioral changes implicated in schizophrenia, bipolar and depressive disorders, suggesting that inflammation can be causally involved in the development of abnormal behavior in mice (Hagemeyer et al., 2012; Janova et al., 2018).

While the leading feature of Hereditary Spastic Paraplegia (HSP) is spasticity of the lower limbs due to axon degeneration in the corticospinal tract, there are complicated forms of HSPs, like HSP type 11 (SPG11), that show superimposed clinical and neurological features of high variance (Klebe et al., 2015; Boutry et al., 2019). Cognitive impairment and neuropsychological symptoms including anxiety, learning and memory difficulties, and changes in social behavior (Denora et al., 2009; Wijemanne et al., 2015; Faber et al., 2018; Utz et al., 2022) are usually present in patients with SPG11. We could previously demonstrate that secondary neuroinflammation by the adaptive immune system acts as a robust disease amplifier in a mouse model of SPG11, leading to remarkable aggravation of histopathological and gait alterations (Hörner et al., 2022). In the present study, we aimed to (i) characterize possible neuropsychological symptoms in the corresponding mouse model and (ii) investigate the possibility whether these symptoms are, at least partially, mediated or aggravated by neuroinflammation and may, thus, be treatable by immunomodulators.

We provide evidence that our SPG11 model shows distinct behavioral abnormalities, including abnormal social, impulsivity-like, anxiety-like, and hyperactivity-like behavior, which partially reflects neuropsychological changes seen in patients (Wijemanne et al., 2015; Faber et al., 2018; Utz et al., 2022; Klebe et al., unpublished data). Importantly, we also show that genetically inactivating the adaptive immune system ameliorates distinct disease-related behavioral abnormalities. Translating this into a clinically applicable approach, we show that treatment with the established immunomodulators fingolimod (FTY720) or teriflunomide, that are in use for treatment of MS, leads to an attenuation of distinct behavioral abnormalities. Together with our previous findings, this may be the base for the clinical use of approved immunomodulators in SPG11 patients and, possibly, other patients with complicated forms of HSP.

## Materials and methods

### Animals

All mice were on a *C57BL/6J* genetic background. Animals were group-housed and kept in individually ventilated cages under barrier conditions at the Center of Experimental Molecular Medicine, University of Würzburg with a 14 h/10 h day/night rhythm (<300 lux during day). Mice were group-housed with sex-matched littermates (3–5 mice/cage). The number of mice housed together was comparable among all experimental groups. No aggressive behavior between the individuals was observed. Colonies were maintained at 20–24°C and 40–60% humidity, with free access to food and water. All animal experiments were approved by the local authority of the Government of Lower Franconia, Germany.

*Spg11*-knockout (*Spg11*^−/−^) mice were used as an SPG11 disease model, and age-matched wild-type (wt, *Spg11^+/+^*) littermates were used as control animals (Branchu et al., 2017). *B6J* refers to *C57BL/6 J* mice bought from Charles River Laboratories. Mice of either sex were analyzed for histopathological readout parameters, as we did not detect sex-related differences in *Spg11*^+/+^ or *Spg11*^−/−^ mice. Genotypes were determined by conventional PCR using isolated DNA from ear punch biopsies as previously described (Branchu et al., 2017). In experiments aimed to genetically inactivate the adaptive immune reactions, *Spg11*^−/−^ mice were crossbred with *Rag1*-deficient (*Rag1^−/−^*) mice (Mombaerts et al., 1992), devoid of mature T- and B-lymphocytes, according to previously published protocols (Ip et al., 2006; Groh et al., 2013). *Rag1^−/−^* mice did not display any abnormalities in longevity and body weight under the standardized conditions applied (specific pathogen-free). For behavioral analysis *Spg11*^+/+^, *Spg11*^−/−^, *Spg11*^−/−^*Rag1^−/−^* and *B6J* mice were divided by sex to minimize sex-bias. For each group a minimum of 3–5 animals per genotype and sex were tested (Tables 1, 2).

**Table 1:** Investigated cohorts for behavioral assessment of male mice.

**Male**	**6 months**	**12 months**	**18 months**
***Spg11***^***+/+***^	10	10	10
***Spg11***^***−/−***^	8	8	8
***Spg11***^***−/−***^ ***Rag1***^***−/−***^	5	5	4
***Spg11***^***−/−***^ **+ FTY720**	8	8	8
***Spg11***^***−/−***^ **+ Teri**	7	7	6
***B6J***	3	3	3
***B6J* + FTY720**	4	4	4
***B6J* + Teri**	5	5	5

Mice were assessed longitudinally.

**Table 2:** Investigated cohorts for behavioral assessment of female mice.

**Female**	**6 months**	**12 months**	**18 months**
***Spg11***^***+/+***^	7	7	7
***Spg11***^***−/−***^	6	6	6
***Spg11***^***−/−***^ ***Rag1***^***−/−***^	7	7	7
***Spg11***^***−/−***^ **+ FTY720**	6	6	5
***Spg11***^***−/−***^ **+ Teri**	6	6	6
***B6J***	3	3	3
***B6J* + FTY720**	5	5	5
***B6J* + Teri**	5	5	5

Mice were assessed longitudinally.

### Immunomodulatory treatment

Fingolimod (FTY720; Sigma-Aldrich; SML0700) was dissolved in autoclaved water at 3 μg per milliliter and provided *ad libitum*, corresponding to a dose of 0.5 mg/kg body weight in a mouse with an average body weight of 30 g and an approximate water consumption of 5 ml per day. Teriflunomide (Biorbyt, orb146201) was dissolved in autoclaved drinking water containing 0.6% Tween 80 at 60 μg per milliliter and provided *ad libitum*, corresponding to a dose of 10 mg/kg body weight per day. These concentrations are based on previous animal experiments of our group (Groh et al., 2017; Hörner et al., 2022; Abdelwahab et al., 2023) and other laboratories (Metzler et al., 2008; Merrill et al., 2009), implicating dose conversion scaling (Nair and Jacob, 2016). Similar to previous studies, *Spg11*^−/−^ mice receiving water containing only 0.6% Tween 80 did not show any abnormalities in inflammation, longevity, gross behavior, or body weight (data not shown; Groh et al., 2016, 2017, 2018, 2021a; Hörner et al., 2022). Autoclaved drinking water without the compounds was provided to non-treated controls. The water with or without compounds was changed weekly. Mice were treated for 450 days, from 3 months of age until the end of the last series of tests and monitored daily regarding defined burden criteria and phenotypic abnormalities. No obvious side effects were detectable during treatment.

### Behavioral evaluation

**Figure 1:**
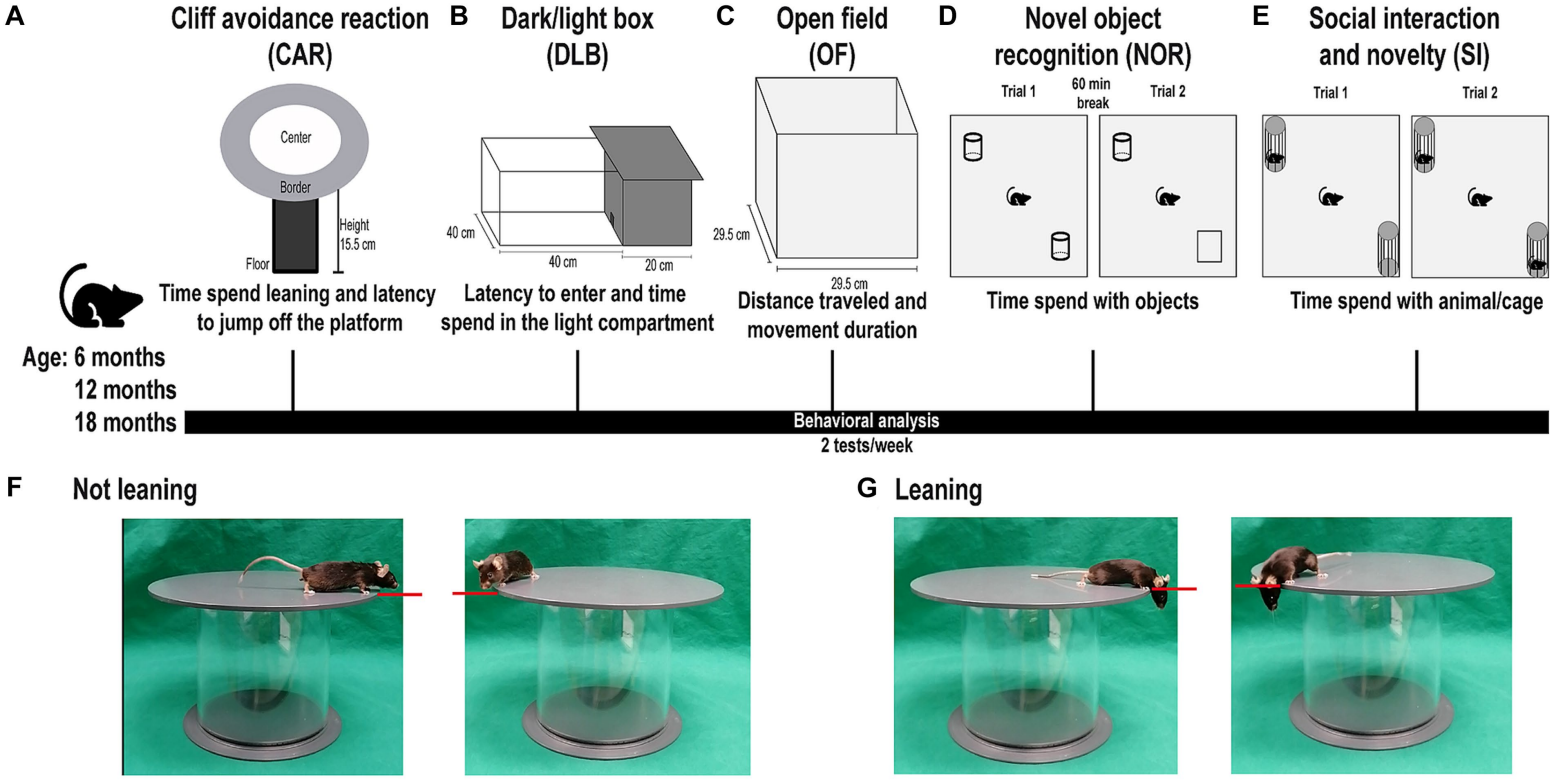
Behavioral test battery for mice. Representative image of the behavioral test battery mice were subjected to in the following order: cliff avoidance reaction (CAR), dark/light box (DLB) analysis, open field (OF) analysis, novel object recognition (NOR), and social interaction and novelty (SI) analysis at the age of 6, 12, and 18 months. The order is structured from less stress-inducing to more stress-inducing tests. Each week a maximum of two tests was performed with a minimum two-day break between tests. **(A)** Graphic depiction of CAR analysis. The platform is approximately 15.5 cm high, non-transparent, and divided into a center (white) and a border (gray) zone. A mouse is placed in the middle of the platform and the time spend leaning and latency to jump off the platform is measured. **(B)** Graphic depiction of DLB analysis. The DLB consists of a light compartment (<300 lx) and a dark compartment, connected by a doorway through which the mouse can walk freely. A mouse is placed in the dark compartment and the latency to enter, and time spend in the light compartment is measured. **(C)** Graphic depiction of OF analysis. A mouse is placed in a wooden and non-transparent OF box. The distance traveled and movement duration of mice is measured. **(D)** Graphic depiction of NOR analysis. A mouse is placed in an OF box, containing two identical objects. After a 60-min break, one object is replaced by an unfamiliar object of similar proportions and the mouse is placed back into the box. The time mice spend with the objects is measured. **(E)** Graphic depiction of SI analysis. A mouse is placed in an OF box containing two small cages. In the social interest trial (trial 1), one cage contains an unfamiliar mouse, while the other cage remains empty. In the social novelty trial (trial 2) a second unfamiliar mouse is placed in the empty cage. The time mice spend with the animal/cage is measured. **(F)** Representative images of mice not leaning over the edge of the CAR platform: A horizontal axis (red line) defines if a mouse is leaning. If the eyes of the mouse do not cross the horizontal axis of the platform rim the mouse is not considered leaning. **(G)** Representative images of mice leaning over the edge of the CAR platform: If the eyes of the mouse cross the horizontal axis of the platform rim the movement is considered leaning. Note that the order of presentation of tests in the results section is different as there it reflects the relevance of our findings for possible translatability to patients.

A behavioral test battery was designed to longitudinally investigate mice (Figure 1). The test battery consisted of the cliff avoidance reaction (CAR) analysis for investigation of impulsivity-like behavior (Figure 1A), the dark/light box (DLB) analysis for investigation of anxiety-like behavior (Figure 1B), the open field (OF) analysis for investigation of exploratory and hyperactivity-like behavior (Figure 1C), the novel object recognition (NOR) analysis for investigation of memory and recognition (Figure 1D), and the social interaction and novelty (SI) analysis for investigation of social interest and novelty behavior (Figure 1E). The tests were performed starting with less stress-inducing tests, followed by more stress-inducing tests, while in the results section, the order of presentation reflects the relevance of our findings for possible translatability to patients. Two tests per week were performed, with at least two and a maximum of four test-free days in between. All tests were recorded using a camera and evaluated using the EthoVisionXT (Noldus Information Technology) video tracking software v.116. All tests were performed in a room with only the investigator present. Mice were placed in the testing room to habituate 30 min prior to each test. Between each trial, arenas, objects and/or cages, were cleaned using 10% ethanol and the arena was aired out for approximately 3 min to minimize behavioral disturbance by olfaction. No more than two tests per week were performed to minimize stress bias. The investigator was unaware of genotypes and possible treatment of the mice during testing.

### Cliff avoidance reaction

To assess impulsivity-like behavior, the CAR analysis was used. CAR was evaluated by using a round, non-transparent, plastic platform (diameter: 25 cm; thickness: 2 cm), supported by a plastic rod (height: 15.5 cm) (Figure 1A). The platform was divided into a center zone (diameter: 20 cm) and a border zone (width: 2.5 cm). To initiate the test, one mouse was gently placed into the center of the platform. The test lasted 10 min. The investigator recorded the latency to jump off the platform and the time the mouse spent leaning over the edge of the platform. A behavior was defined as “jumping behavior,” when a mouse voluntarily jumped off the platform. If a mouse jumped off the platform during the test, it was gently placed back in the middle of the platform. The definition of “leaning over the edge of the platform” is depicted in Figure 1. A behavior was considered “leaning over the edge of the platform” when the eyes of the mouse crossed the horizontal axis of the platform rim (Figures 1F,G).

### Dark/light box analysis

To evaluate anxiety-like behavior in mice, DLB analysis was used. The DLB consists of a dark compartment (20 cm × 40 cm), and a light compartment (40 cm × 40 cm; <300 lx), connected by a small doorway (Figure 1B). To begin the experiment, one mouse was placed in a random corner of the dark compartment of the box, facing the wall and away from the doorway. Corners were randomly alternated between mice to minimize bias. The mouse was allowed to explore freely and was recorded in the light, but not dark compartment. After 10 min, the mouse was taken out of the arena, disregarding if it had entered the light compartment or not. The investigator recorded the time until entering the light compartment of the arena and the time spent in the light compartment.

### Open field analysis

To evaluate the general exploratory and hyperactivity-like behavior of mice, the OF analysis was used. To begin the experiment, one mouse was gently placed in a corner of a white, non-transparent, wooden box (29.5 cm × 29.5 cm), facing the wall (Figure 1C). The corners were randomly selected and alternated between different mice. One trial lasted a total of 10 min. Total walking distance and movement duration was assessed by the EthoVisionXT software.

### Novel object recognition

For assessment of non-spatial learning and recognition, the NOR analysis was used as previously described (Groh et al., 2021b). The test was performed in an OF box (29.5 cm × 29.5 cm), which was divided into quadrants of the same size. For the training trial, two identical objects were placed in opposite quadrants of the arena (Figure 1D). The quadrants were randomly alternated between tests to minimize bias. To start the experiment, one mouse was gently placed into a corner of a quadrant not containing an object, facing the wall. The corners were randomly selected and alternated between mice. After the training trial, the mouse was taken out of the arena and placed back in its home cage for 60 min. For the test trial, one of the objects was replaced by an unfamiliar object that was of similar dimensions and colors but yielded enough discrimination properties and did not show spontaneous preference in prior testing (data not shown). The mouse was placed back into a corner of the arena, in a quadrant not containing an object, facing the wall. During both trials, the mouse was allowed to explore freely for 10 min while being recorded. In both trials, the time spend actively sniffing or touching each object was recorded by the investigator. The object preference was calculated for training and test trial: [time spent with object 1/time spent with object 2] × 100. In the test trial object 1 refers to the unfamiliar object.

### Social interaction and novelty analysis

For assessment of social interaction and novelty behavior, the SI analysis was used. Two small, identical cages were placed in an OF box (29.5 cm × 29.5 cm) in diagonal corners of the arena (Figure 1E). The corners containing the cages were chosen randomly and alternated between different tests to minimize bias. In the social interest trial (trial 1), one of the cages contained a sex-matched mouse that was unfamiliar to the test mouse. The cage containing the mouse was chosen randomly and alternated between tests to minimize bias. The test mouse was gently placed in one of the corners of the arena, not containing a cage, facing the wall. The corners were alternated randomly between mice. The social novelty trial (trial 2) was carried out directly following the social interest trial (trial 1). A second, unknown, sex-matched mouse was placed in the empty cage of the arena. To start the social novelty trial (trial 2), the test mouse was gently placed in a corner, not containing a cage, facing the wall. The test mouse was allowed to explore freely for 10 min. The time spent with the unfamiliar mice or the empty cage (direct interaction; sniffing or touching) was recorded by the investigator. The social preference was calculated for both trials. Social interest preference (trial 1): [time spent with animal 1/time spent with empty cage] × 100. Social novelty preference (trial 2): [time spent with animal 2/time spent with animal1] × 100. Animal 1 being the first unfamiliar animal, and animal 2 being the second unfamiliar animal. When the unfamiliar mice were taken out of the cages, the cages were cleaned using 10% ethanol and given time to air out for approximately 3 min. The mice used as unfamiliar mice were placed in the cages for 5 min each day starting 3 days prior to testing day for habituation.

### Dissection and processing of nervous tissue

Mice were euthanized using CO_2_ (according to guidelines by the State Office of Health and Social Affairs Berlin) and transcardially perfused with phosphate-buffered saline (PBS) containing heparin followed by 2% paraformaldehyde (PFA) in PBS. Subsequently, tissue was harvested, post-fixed, dehydrated and processed as previously described (Groh et al., 2016). Before embedding of the brain, the olfactory bulb was removed, the cerebrum and cerebellum were separated at defined positions and embedded in TissueTek ® O.C.T.™ compound.

### Histochemistry and immunofluorescence

Immunohistochemistry was performed on 40 μm thick coronal cerebellum cryo-sections (Bregma −6.00 to −6.30 mm). Sections were washed and blocked using 5% bovine serum albumin/1% normal goat serum/0.3% Triton X100 in PBS. Sections were incubated over night at 4°C with the following primary antibody: mouse anti-calbindin (1: 500, Swant Inc., 300). Primary antibody binding was visualized using a fluorescently labeled anti-mouse secondary antibody (1:300, Dianova). Fluorescence images were acquired using an Axio Imager M2 microscope (Zeiss; Objective: 20x/0.8 Ph2 M27) with ApoTome.2 structured illumination equipment, attached Axiocam cameras and corresponding software (ZEN v.2.3 blue edition). Images were minimally processed (rotation and cropping) using Photoshop CS6 (Adobe). For quantification of Purkinje cells (PC), calbindin^+^ cells were counted in at in at least three coronal cerebellum cryo-sections for each animal and related to the area of the section using the cell counter plugin in ImageJ (National Institutes of Health, Bethesda USA).

### Experimental design and statistical analysis

Quantifications and clinical analyses were performed by investigators unaware of the genotype and potential treatments of mice, by assigning uniquely coded labels to animals. Mice were randomly placed into experimental and control groups according to genotyping results and by using a random generator.^1^ For biometrical sample size estimation, the G*Power program (version 3.1.3) was used (Faul et al., 2007). Calculation of the appropriate sample size groups was performed in *a priori* power analysis by comparing the mean of three to four groups with a defined adequate power of 0.8 (1-beta-error) and an *α*-error of 0.05. To determine the prespecified effect size *d*, previously published data were considered comparable reference values (Groh et al., 2017, 2021a; Hörner et al., 2022). Statistical analysis was performed using Prism 8 (GraphPad). The Shapiro–Wilk test was used to assess data sets for normal distribution and the *F* test was used to check for variance homology. If more than two groups or multiple timepoints (behavioral analysis) were compared, differences were evaluated by one-way ANOVA, followed by Sidak’s *post-hoc* test. Significance levels are indicated by asterisks (*) and considered significant according to the following scheme: *p < 0.05; **p < 0.01; ***p < 0.001. Measurements and quantifications are shown as individual values (circles, squares, triangles = mean value of one mouse) and mean ± standard deviation (SD). Graphs were generated using Prism 8.

## Results

### *Spg11^−/−^* mice display distinct behavioral abnormalities.

Patients suffering from complex HSP forms often present with distinct neuropsychological features, including anxiety, hyperactivity, impulsivity, learning and memory difficulties, and changes in social behavior (Osmolak et al., 2012; Wijemanne et al., 2015; Faber et al., 2018; Utz et al., 2022). We here examined whether similar behavioral alterations are detectable in *Spg11*^−/−^ mice by using a behavioral test battery comprising tests for social interaction and novelty behavior, novel object recognition, general exploratory, and hyperactivity-like behavior, anxiety-like behavior, and impulsivity-like behavior (Figure 1).

**Figure 2:**
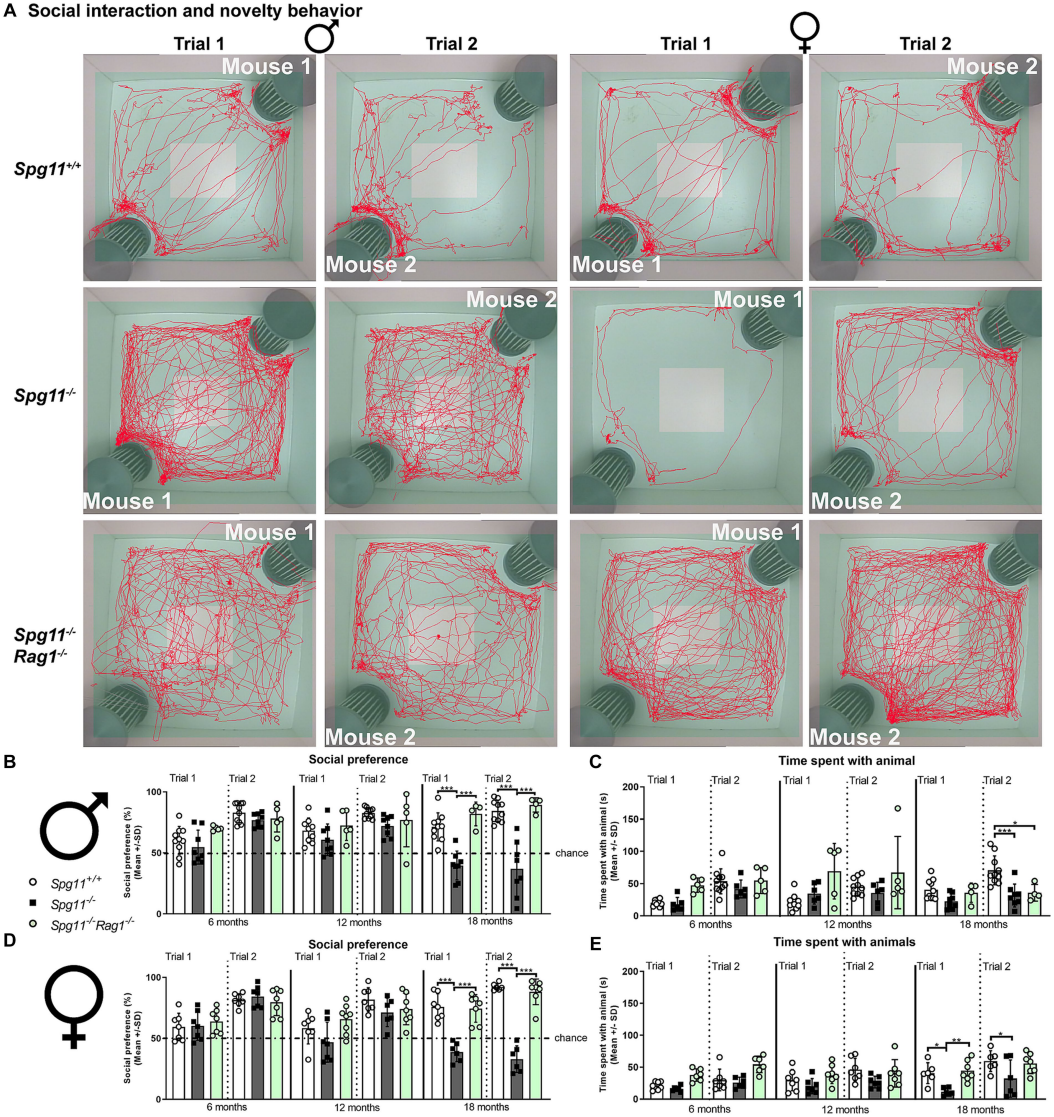
*Spg11^−/−^* mice show social abnormalities, which are ameliorated by *Rag1*-deficiency. **(A)** Representative images of social interest (trial 1, 1st, and 3rd column) and novelty (trial 2, 2nd, and 4th column) analysis of 18-month-old male (1st and 2nd column) and female (3rd and 4th column) *Spg11*^+/+^ (top row), *Spg11*^−/−^ mice (2nd row), and *Spg11*^−/−^*Rag1^−/−^* mice (bottom row). Light square indicates the center zone of the arena, green area indicates the border zone. Red line represents the walking track. Mouse 1 indicates the cage in which the first unfamiliar mouse is placed, mouse 2 in which the second unfamiliar mouse is placed. Empty indicates the empty cage. **(B)** Social interest and novelty preference of 6-, 12-, and 18-month-old male mice. Male *Spg11*^−/−^ mice show lower social interest and novelty preference compared to wt mice at 18 months, which is restored to wt levels by *Rag1*-deficiency [*F*(17, 117) = 12.14, *p* < 0.0001]. **(C)** Time spent with unfamiliar animals of male mice at 6, 12, and 18 months. Male *Spg11*^−/−^ mice less time with the unfamiliar animals during the social novelty trial (trial 2) compared to wt littermates at 18 months. This is not altered by *Rag1*-deficiency [*F*(17, 110) = 5.077, *p* < 0.0001]. **(D)** Social interest and novelty preference of 6-, 12-, and 18-month-old female mice. Female *Spg11*^−/−^ mice show lower social interest and novelty preference compared to their wt littermates at 18 months, which is restored to wt levels by *Rag1*-deficiency [*F*(23, 131) = 10.55, *p* < 0.0001]. **(E)** Time spent with unfamiliar animals of female mice at 6, 12, and 18 months. Female *Spg11*^−/−^ mice spend less time with the unfamiliar animals compared to their wt littermates in the social interest trial (trial 1) at 18 months, and in the social novelty trial (trial 2) at 12 and 18 months. *Rag1*-deficiency increases the time female *Spg11*^−/−^ mice spend with the unfamiliar animals in the social interest (trial 1) and novelty trial (trial 2) [*F*(17, 94) = 4.875, *p* < 0.0001]. Chance indicates equal time spent with the empty cage/animals (50%). Error bars represent standard deviations (circles, squares = value of one mouse). Significance *Spg11^−/−^* compared to *Spg11^+/+^* and *Spg11^−/−^Rag1^−/−^* compared to *Spg11^+/+^* and *Spg11^−/−^* mice is determined by one-way ANOVA and Sidak’s *post hoc* test (*p < 0.05, **p < 0.01, ***p < 0.001).

The social interaction and novelty (SI) analysis comprises a social interest (trial 1) and novelty trial (trial 2) (Figure 1E). In both trials, the time the test mice spend directly sniffing or touching the unfamiliar mice or the empty cage is recorded (Figure 1E). Male and female *Spg11*^−/−^ mice showed a significant reduction of social interest and novelty preference compared to wt mice at 18 months of age (Figures 2A,B,D). Furthermore, *Spg11*^−/−^ mice of both sexes spent less time with the unfamiliar animals during the social novelty trial (trial 2) compared to their wt littermates (Figures 2C,E), reflecting abnormal social behavior. Interestingly, while male *Spg11*^−/−^ mice showed a reduction in time spent with the unfamiliar animal only at 18 months of age, female *Spg11*^−/−^ mice spent less time with the unfamiliar animals already at 12 months, though the social novelty preference was still comparable to wt mice (Figures 2A–E). Of note, the novel object recognition, a measure of non-spatial memory and recognition (Figure 1D), was not altered in male or female *Spg11*^−/−^ mice at any investigated age (Supplementary Figures S1a,b).

**Figure 3:**
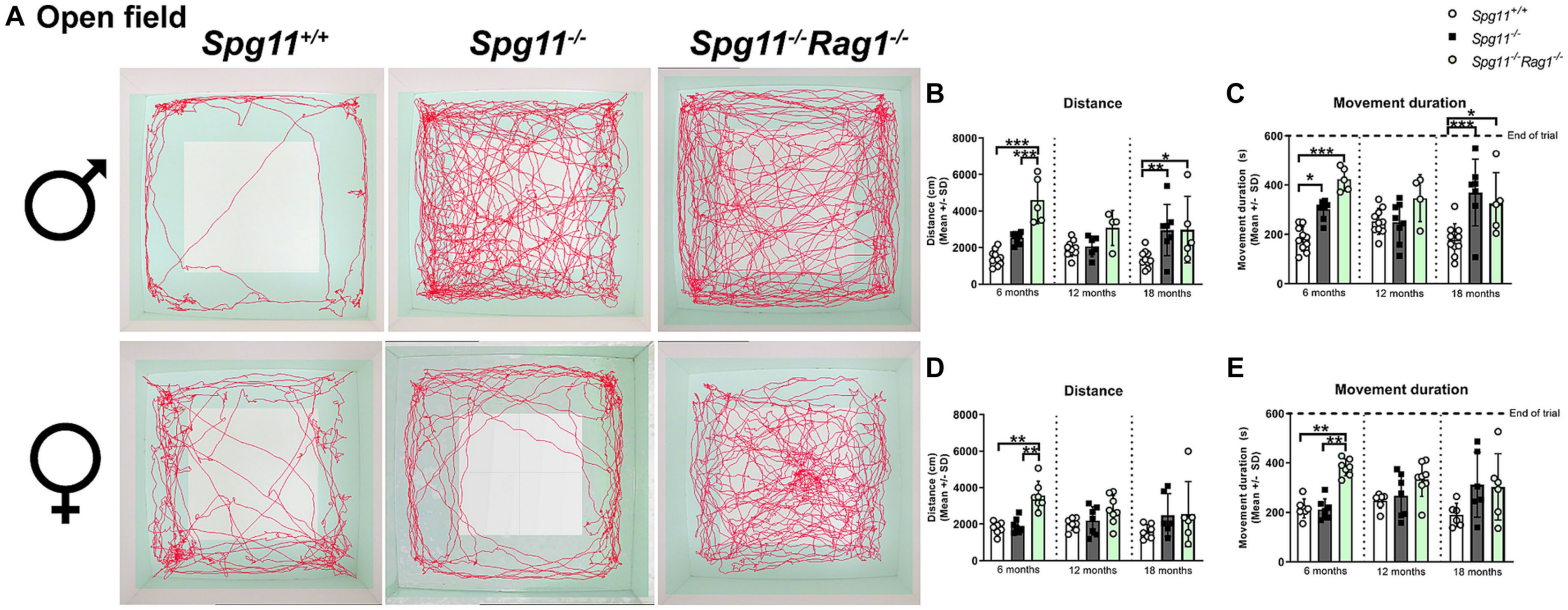
*Spg11^−/−^* mice show hyperactivity-like behavior, which is not ameliorated by *Rag1*-deficiency. **(A)** Representative images of OF analysis of 18-month-old male (top row) and female (bottom row) *Spg11^+/+^*, *Spg11^−/−^*, and *Spg11^−/−^Rag1^−/−^* mice. Light square indicates the center zone of the arena, green area indicates the border zone. Red line represents the walking track. **(B)** Male *Spg11^−/−^* mice walk a greater distance and **(C)** show a higher movement duration than their wt littermates at 6 and 18 months. This is not reduced by *Rag1*-deficiency. Of note, male *Rag1*-deficienct *Spg11^−/−^* mice walk a greater distance and show a higher movement duration compared to wt and *Spg11^−/−^* mice at 6 months [distance: *F*(11, 79) = 6.021, *p* < 0.0001; movement duration: *F*(11, 81) = 5.389, *p* < 0.0001]. **(D)** Female *Spg11^−/−^* mice walk a greater distance and **(E)** show a higher movement duration compared to their wt littermates at 18 months. This is not reduced by *Rag1*-deficiency. Of note, female *Rag1*-deficienct *Spg11^−/−^* mice walk a greater distance and show a higher movement duration compared to wt and *Spg11^−/−^* mice at 6 months [distance: *F*(11, 65) = 3.037, *p* = 0.0025; movement duration: *F*(11, 61) = 3.455, *p* = 0.0009]. Error bars represent standard deviations (circles, squares = value of one mouse). Significance of *Spg11^−/−^* compared to *Spg11^+/+^* mice and of *Spg11^−/−^Rag1^−/−^* compared to *Spg11^+/+^* and *Spg11^−/−^* mice is determined by one-way ANOVA and Sidak’s *post hoc* test (*p < 0.05, **p < 0.01, ***p < 0.001).

To investigate exploratory and hyperactivity-like behavior, we performed the open field (OF) analysis, measuring the distance mice traveled and their movement duration (Figure 1C). Male *Spg11*^−/−^ mice walked longer distances and showed a higher movement duration at 6 and 18 months of age compared to wt mice (Figures 3A–C), indicating hyperactivity-like behavior. Of note, the time male *Spg11*^−/−^ mice spent in the center zone of the arena remained unaffected (data not shown). In contrast, female *Spg11*^−/−^ showed an increased walking distance and movement duration only at 18 months of age (Figures 3A,D,E) and the center time remained unaffected compared to wt mice at all ages investigated (data not shown).

**Figure 4:**
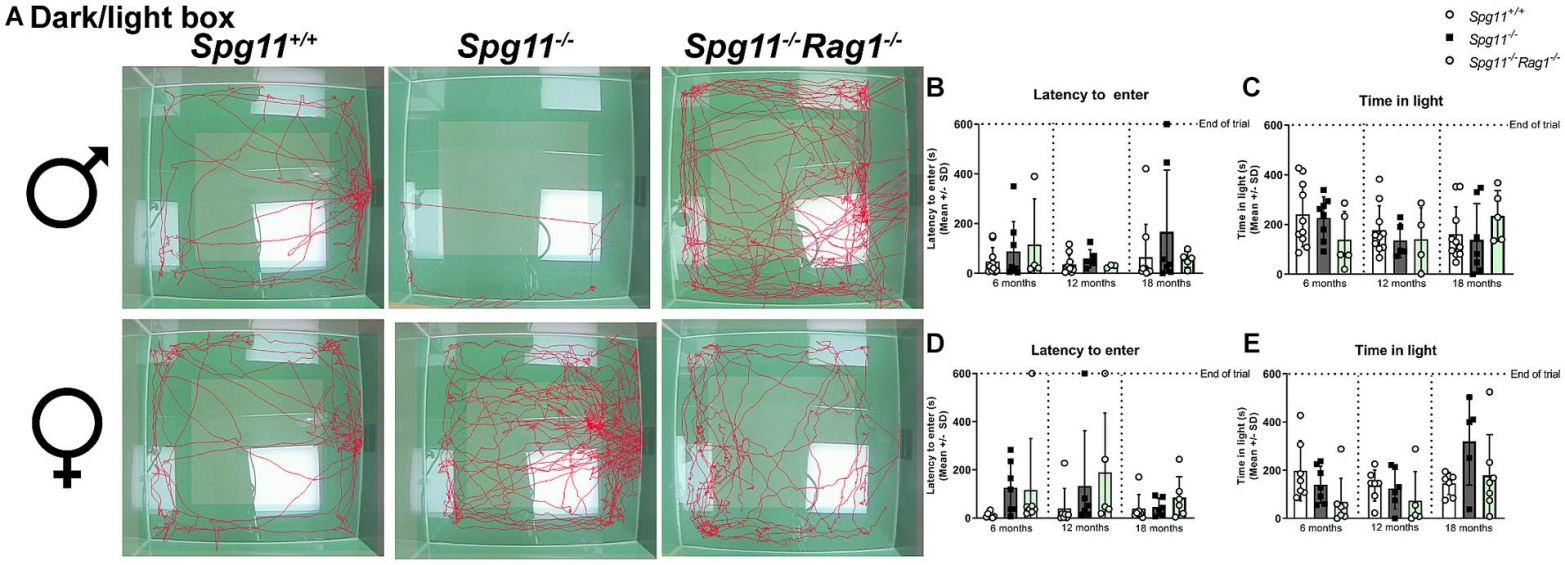
Female *Spg11^−/−^* mice show reduced anxiety-like behavior, which is restored by *Rag1*-deficiency. **(A)** Representative images of DLB analysis of 18-month-old male (top row) and female (bottom row) *Spg11^+/+^*, *Spg11^−/−^*, and *Spg11^−/−^Rag1^−/−^* mice. Light square indicates the center zone of the arena. Red line represents the walking track. **(B)** Male *Spg11*^−/−^ or *Spg11^−/–^Rag1^−/−^* mice do not show an altered latency to enter the light compartment, or **(C)** time spend in the light compartment compared to wt mice at any investigated age [latency to enter: *F*(11, 73) = 1.675, *p* = 0.0964; time spend in light compartment: *F*(11, 74) = 2.084, *p* = 0.0319]. **(D)** Female *Spg11*^−/−^ or *Spg11^−/−^Rag1^−/−^* mice do not show an altered latency to enter the light compartment at any investigated age [*F*(11, 61) = 1.098, *p* = 0.3785]. **(E)** Female *Spg11*^−/−^ show a strong tendency towards more time spend in the light compartment than their wt littermates at 18 months. This is restored by *Rag1*-deficiency [*F*(11, 62) = 2.095, *p* = 0.0339]. Error bars represent standard deviations (circles, squares = mean value of one mouse). Significance of *Spg11^−/−^* compared to *Spg11^+/+^* and *Spg11^−/−^Rag1^−/−^* compared to *Spg11^+/+^* and *Spg11^−/−^* mice is determined by one-way ANOVA and Sidak’s *post hoc* test.

To assess the anxiety-like behavior in this mouse model, we performed the commonly used dark/light box (DLB) analysis, recording the latency to enter and the time mice spend in the light compartment (Figure 1B). While we did not see differences in the behavior of male *Spg11*^−/−^ mice compared to their wt littermates (Figures 4A–C), we detected distinct differences between female *Spg11*^−/−^ and *Spg11*^+/+^ mice at 18 months of age (Figures 4A,D,E). While female *Spg11*^−/−^ mice did not show an altered latency to enter the light compartment, they showed a strong tendency to spend more time in the light compartment than their wt littermates (Figures 4D,E), indicating reduced anxiety-like behavior.

**Figure 5:**
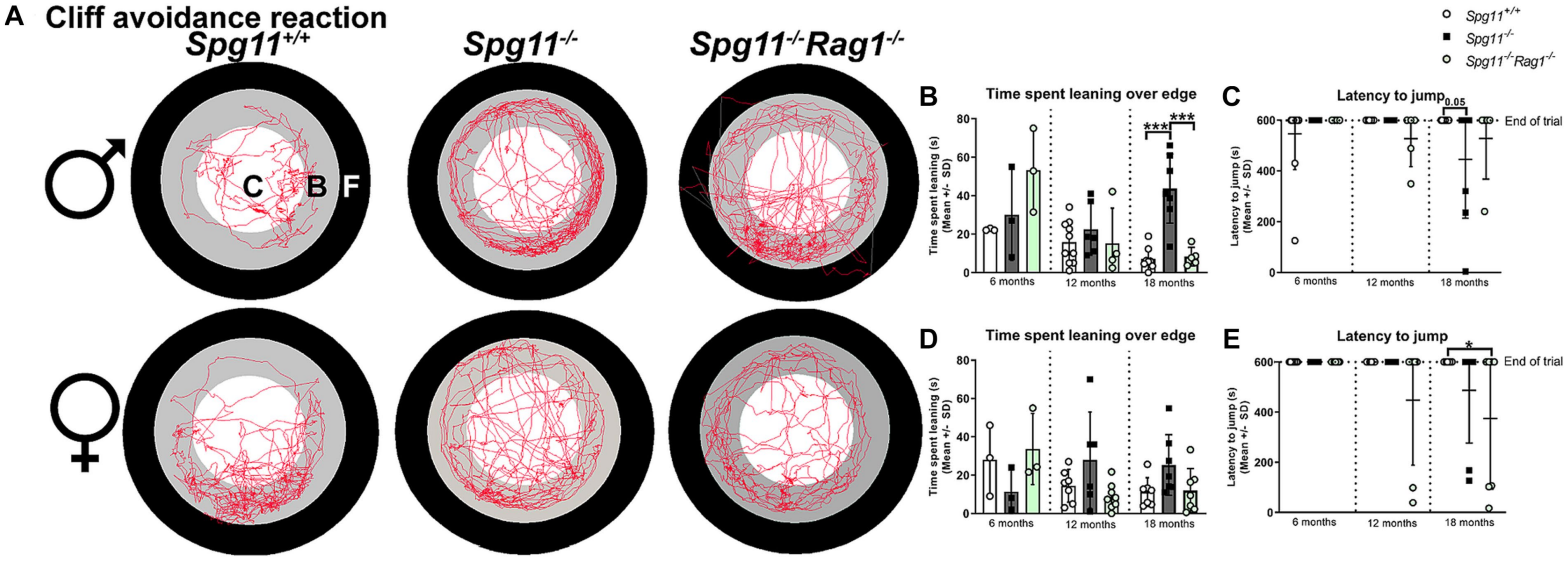
*Spg11^−/−^* mice show impulsivity-like behavior, which is partially reduced by *Rag1*-deficiency. **(A)** Representative images of CAR analysis of 18-month-old male (top row) and female (bottom row) *Spg11^+/+^*, *Spg11^−/−^*, and *Spg11^−/−^Rag1^−/−^* mice. Red line represents the walking track (C = center zone, white; B = border zone, grey; F = floor zone, black). **(B)** Male *Spg11^−/−^* mice spend more time leaning over the edge of the platform at 18 months. This is reduced by *Rag1*-deficiency [*F*(11, 55) = 8.024, *p* < 0.0001]. **(C)** Male *Spg11^−/−^* and *Spg11^−/−^Rag1^−/−^* mice show a lower latency to jump off the platform compared to their wt littermates at 18 months [*F*(8, 54) = 1.643, *p* = 0.1343]. **(D)** Female *Spg11^−/−^* show a tendency to spend more time leaning over the edge of the platform at 18 months. This is reduced by *Rag1*-deficiency [*F*(8, 42) = 2.219, *p* = 0.0451]. **(E)** Female *Spg11^−/−^* and *Spg11^−/−^Rag1^−/−^* mice show a lower latency to jump off the platform compared to their wt littermates at 18 months [*F*(8, 59) = 2.684, *p* = 0.0138]. Error bars represent standard deviations (circles, squares = value of one mouse). Significance of *Spg11^−/−^* compared to *Spg11^+/+^* and *Spg11^−/−^Rag1^−/−^* compared to *Spg11^+/+^* and *Spg11^−/−^* mice is determined by one-way ANOVA and Sidak’s *post hoc* test (*p < 0.05, ***p < 0.001).

To assess the impulsivity-like behavior of *Spg11^−/−^* mice, we used the cliff avoidance reaction (CAR) analysis (Figure 1A). Here, the time mice spend leaning over the edge of the platform and the latency to jump off the platform is recorded (Figures 1A,F,G). Male *Spg11^−/−^* mice spent more time leaning over the edge of the platform at 18 months compared to wt mice (Figures 5A,B). At the same age, they showed a reduced latency to jump off the platform compared to wt littermates, indicating impulsivity-like behavior (Figure 5C). Interestingly, female *Spg11^−/−^* mice showed only a tendency to spend more time leaning over the edge and to jump off the platform compared to their wt littermates at 18 months (Figures 5A,D,E). Wt mice of either sex rarely jumped off the platform at any investigated age (Figures 5C,E). Of note, *Spg11^−/−^* did not show an increased risk to involuntarily fall off the platform compared to wt littermates.

In summary, we here demonstrate that male and female *Spg11*^−/−^ mice show distinct behavioral abnormalities, comprising reduced social interest and novelty behavior, as well as hyperactivity-like and impulsivity-like behavior. Additionally, female *Spg11*^−/−^ mice show reduced anxiety-like behavior. Non-spatial memory and recognition remains unaffected in *Spg11*^−/−^ mice. Importantly, the detected social abnormalities were not due to a general reduction of exploratory behavior or recognition, or increased anxiety-like behavior. This suggests that this mouse model displays the neuropsychological characteristics of SPG11 to a large degree.

### Genetic inactivation of the adaptive immune system ameliorates behavioral abnormalities of *Spg11^−/−^* mice

Based on our previous observation that adaptive immune reactions substantially amplify neuropathological features and disturbed gait coordination in this mouse model for SPG11 (Hörner et al., 2022), we here investigated whether immune reactions could mediate or aggravate behavioral abnormalities as well. As a proof-of-principle approach, we evaluated the putative impact of lymphocytes in *Spg11*^−/−^ mice by crossbreeding them with *Rag1*-deficient (*Rag1*^−/−^) mice, lacking mature T- and B-lymphocytes (Mombaerts et al., 1992). We found that in *Spg11^−/−^* mice, *Rag1*-deficiency restored the social interest and novelty preference of male *Spg11^−/−^* mice to wt levels at 18 months of age (Figures 2A,B). Interestingly, even though the social novelty preference (trial 2) was restored, the time spent with the unfamiliar animals during this trial remained reduced (Figure 2C). In female *Spg11*^−/−^ mice, *Rag1*-deficiency restored the social interest and novelty preference (Figures 2A,D), and increased the time 18-month-old female knockout mice spent with the unfamiliar animals in both trials (Figure 2E). Thus, abnormalities in the social behavior of female *Spg11*^−/−^ mice were completely prevented. *Rag1*-deficiency did not affect the social interest and novelty behavior of male or female *Spg11*^+/+^ mice at any investigated age (Supplementary Figures S2a–d). Of note, the novel object recognition of male or female wt or knockout mice was not altered by *Rag1*-deficiency (Supplementary Figures S1a,b, S2e,f).

*Rag1*-deficiency did not reduce hyperactivity-like behavior of male or female *Spg11*^−/−^ mice, as revealed by OF analysis (Figures 3A–E). Surprisingly, male and female *Spg11*^−/−^*Rag1*^−/−^ mice walked a greater distance and showed a higher movement duration than *Spg11*^−/−^ mice at 6 months of age (Figures 3A–E). While the walking distance and movement duration increased in *Spg11*^−/−^ mice between 6 and 18 months, it remained unchanged in *Rag1*-deficient *Spg11*^−/−^ mice, leading to similar values at 18 months (Figures 3A–E). *Rag1*-deficiency did not alter the exploratory behavior of male or female *Spg11*^+/+^ mice at any investigated age (Supplementary Figures S2g–j).

Regarding anxiety-related parameters, *Rag1*-deficiency did not lead to any behavioral changes of male *Spg11*^−/−^ mice in the DLB (Figures 4A–C). While *Rag1*-deficiency did not alter the latency to enter the light compartment of female *Spg11^−/−^* mice (Figures 4A,D), it led to a tendency towards less time spent in the light compartment, a parameter typically increased in 18-month-old female *Spg11*^−/−^ mice (Figures 4A,E). Of note, *Rag1*-deficiency led to an increased latency to enter and a tendency to spend less time in the light compartment in male and female (Supplementary Figures S2k–n).

In the CAR, *Rag1*-deficiency reduced the time spent leaning over the edge of the platform, typically increased in 18-month-old male *Spg11*^−/−^ mice (Figures 5A,B), and showed the same tendency for female *Spg11^−/−^Rag1^−/−^* mice (Figures 5A,D). However, it did not ameliorate the latency to jump off the platform of male or female *Spg11^−/−^* mice (Figures 5C,E). While male *Rag1*-deficient *Spg11*^+/+^ mice spent more time leaning over the platform at 6 months, all other parameters remained unaffected at all investigated ages (Supplementary Figures S2o–r).

The previously presented data indicate that neuropsychological abnormalities are, at least partially, driven by the adaptive immune system. Complementary to our previous study, these findings show that secondary inflammation does not only contribute to neuropathological and gait alterations (Hörner et al., 2022) but also to behavioral abnormalities.

### Treatment with fingolimod or teriflunomide ameliorates behavioral abnormalities of *Spg11^−/−^* mice

Since we could show that behavioral abnormalities of *Spg11^−/−^* mice are, at least partially, driven by the adaptive immune system, we investigated whether immunomodulatory treatment, as a more clinically relevant approach, would lead to similar beneficial effects.

**Figure 6:**
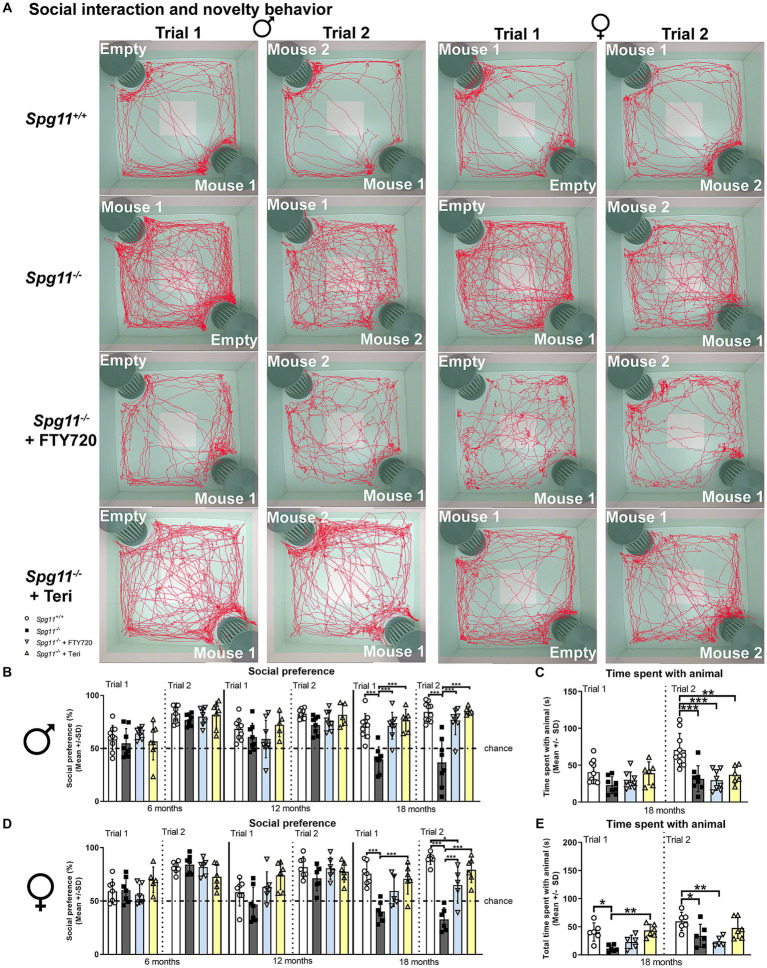
Treatment with fingolimod or teriflunomide ameliorates social abnormalities of *Spg11^−/−^* mice. **(A)** Representative images of social interest (trial 1; 1st and 3rd column) and novelty (trial 2; 2nd and 4th column) analysis of 18-month-old male (1st and 2nd column) and female (3rd and 4th column) *Spg11*^+/+^ (top row), *Spg11*^−/−^ mice (2nd row), and *Spg11*^−/−^ mice treated with fingolimod (FTY720) (3rd row) or teriflunomide (bottom row). Light square indicates the center zone of the arena, green area indicates the border zone. Red line represents the walking track. Mouse 1 indicates the cage in which the first unfamiliar mouse is placed, mouse 2 in which the second unfamiliar mouse is placed. Empty indicates the empty cage. **(B)** Social preference of male *Spg11*^+/+^, *Spg11^−/−^* and treated *Spg11^−/−^* mice at 6, 12, and 18 months. Treatment with either immunomodulator restores social interest and novelty preference of 18-month-old male *Spg11*^−/−^ mice [social preference: *F*(11, 82) = 4.826, *p* < 0.0001; time spent with animal: *F*(7, 56) = 8.078, *p* < 0.0001]. **(C)** Treatment does not increase the time male *Spg11*^−/−^ mice spend with the unfamiliar animals in the social novelty trial (trial 2). **(D)** Social preference of female *Spg11*^+/+^, *Spg11^−/−^* or treated *Spg11^−/−^* mice at 6, 12, and 18 months. Treatment with either immunomodulator increases the social interest and novelty preference of 18-month-old female *Spg11*^−/−^ mice, while the effect of teriflunomide treatment is more pronounced [*F*(23, 126) = 9.881, *p* < 0.0001]. **(E)** Treatment with teriflunomide, but not fingolimod, increases the time female *Spg11*^−/−^ mice spend with the unfamiliar animal in the social interest trial (trial 1) and shows the same tendency in the social novelty trial (trial 2) at 18 months [*F*(7, 38) = 6.643, *p* < 0.0001]. Chance indicates equal time spent with the empty cage/animals (50%). Error bars represent the standard deviations (circles, squares, triangles = mean value of one mouse). Significance of treated knockout mice compared to *Spg11^+/+^* and untreated *Spg11^−/−^* mice is determined by one-way ANOVA and Sidak’s *post hoc* test (*p < 0.05, **p < 0.01, ***p < 0.001). Corresponding data from *Spg11^+/+^* and *Spg11^−/−^* mice, as shown in Figure 2, are presented here again.

**Figure 7:**
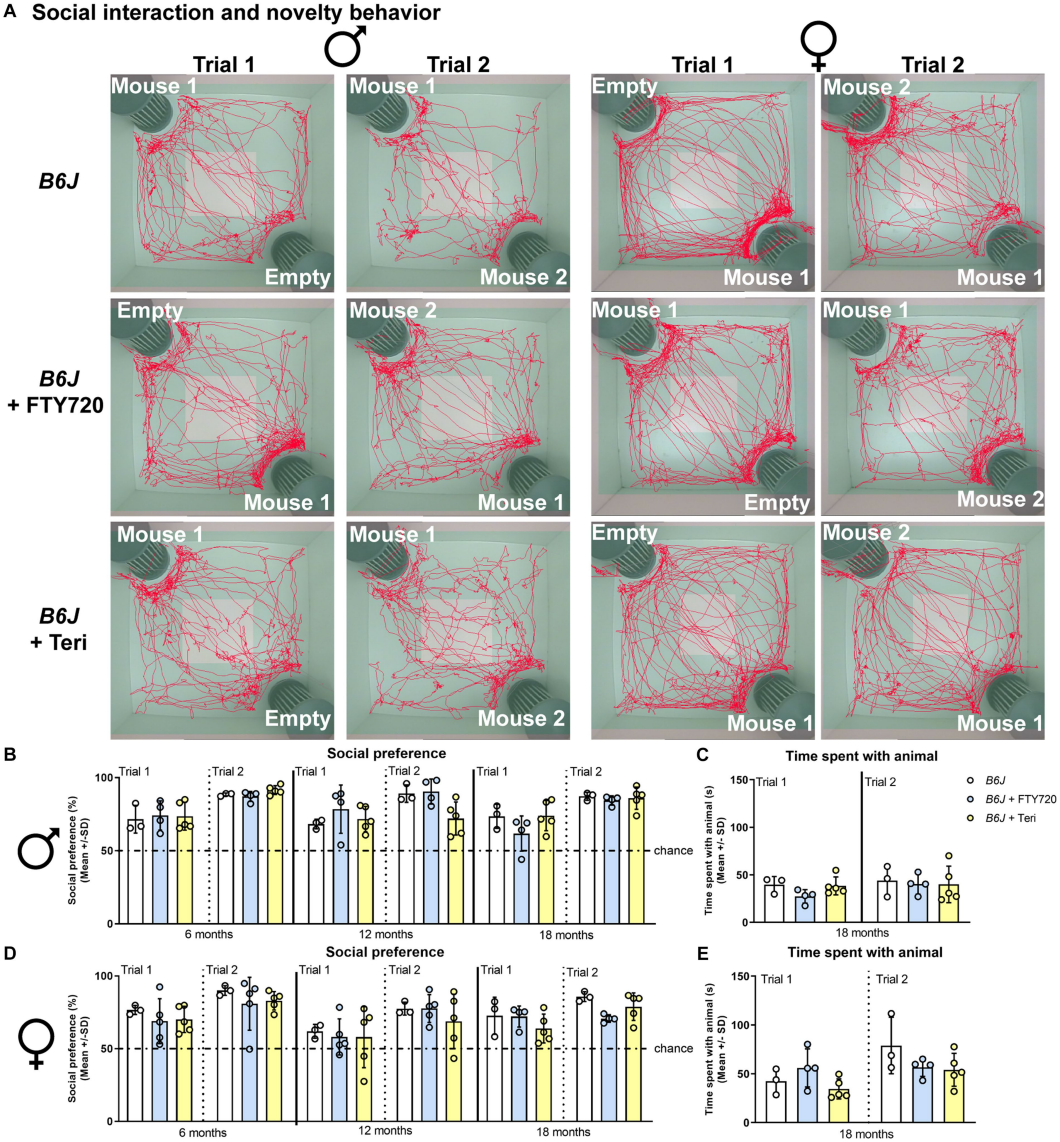
Treatment with fingolimod or teriflunomide does not alter social behavior of *B6J* mice. **(A)** Representative images of social interest (trial 1; 1st and 3rd column) and novelty (trial 2; 2nd and 4th column) analysis of 18-month-old male (1st and 2nd column) and female (3rd and 4th column) *B6J* (top row) and *B6J* mice treated with fingolimod (FTY720) (2nd row) or teriflunomide (bottom row). Light square indicates the center zone of the arena, green area indicates the border zone. Red line represents the walking track. Mouse 1 indicates in which cage the first unfamiliar mouse was placed, mouse 2 in which the second unfamiliar mouse was placed. Empty indicates the empty cage. **(B)** Social preference of *B6J* or treated *B6J* mice at 6-, 12-, and 18- months. Treatment does not alter social interest and novelty preference or **(C)** the time spend with the unfamiliar animals of male *B6J* mice [social preference: *F*(17, 54) = 4.146, *p* < 0.0001; time spent with animal: *F*(5, 18) = 0.7371, *p* = 0.6054]. **(D)** Social preference of female *B6J* or treated *B6J* mice at 6-, 12-, and 18- months. Treatment does not alter social interest and novelty preference, or **(E)** the time spend with the unfamiliar animals of female *B6J* mice [social preference: *F*(17, 58) = 2.382, *p* = 0.0074; time spent with animal: *F*(5, 18) = 3.001, *p* = 0.0384]. Chance indicates equal time spent with the empty cage/animals (50%). Error bars represent standard deviations (circles = value of one mouse). Significance of treated *B6J* mice compared to *B6J* mice is determined by one-way ANOVA and Sidak’s *post hoc* test.

Indeed, we found striking benefits of treatment, starting at 3 months of age, with either fingolimod or teriflunomide on the social behavior of *Spg11^−/−^* mice (Figures 6A–E). Treatment restored social interest (trial 1) and novelty preference (trial 2) of 18-month-old male *Spg11^−/−^* mice to wt levels (Figures 6A,B). Interestingly, and resembling the findings in *Rag1*-deficient *Spg11^−/−^* mice, treatment did not increase the time male *Spg11^−/−^* mice spent with the unfamiliar animals in the social novelty trial at this age (Figure 6C). Remarkably, treatment with teriflunomide did not only restore the social interest (trial 1) and novelty preference (trial 2) of female *Spg11^−/−^* mice to wt levels at 18 months, but treated mice also spent significantly more time with the unfamiliar animal in the social interest trial (trial 1), showing the same tendency in the social novelty trial (trial 2) (Figures 6A,D,E). Treatment with fingolimod also increased the social interest and novelty preference of 18-month-old female *Spg11^−/−^* mice, albeit not to the same degree as treatment with teriflunomide (Figures 6A,D). Interestingly, treatment with fingolimod did not increase the time female *Spg11^−/−^* mice spent with the unfamiliar mice (Figure 6E). The novel object recognition of male or female *Spg11^−/−^* mice was not altered by treatment with either immunomodulator (Supplementary Figures S3a,b). To ensure that treatment alone did not change the behavior of mice, a limited number of *B6J* mice were treated with fingolimod or teriflunomide using the same treatment approach as previously described. Treatment did not alter the social interest (trial 1) or novelty preference (trial 2) of male or female *B6J* mice compared to untreated littermates (Figures 7A–E). The time treated mice spent with the unfamiliar animals also remained unaffected by either treatment (Figures 7A–E). Moreover, the novel object recognition of male or female *B6J* mice was not altered by treatment with either immunomodulator (Supplementary Figures S4a,b).

**Figure 8:**
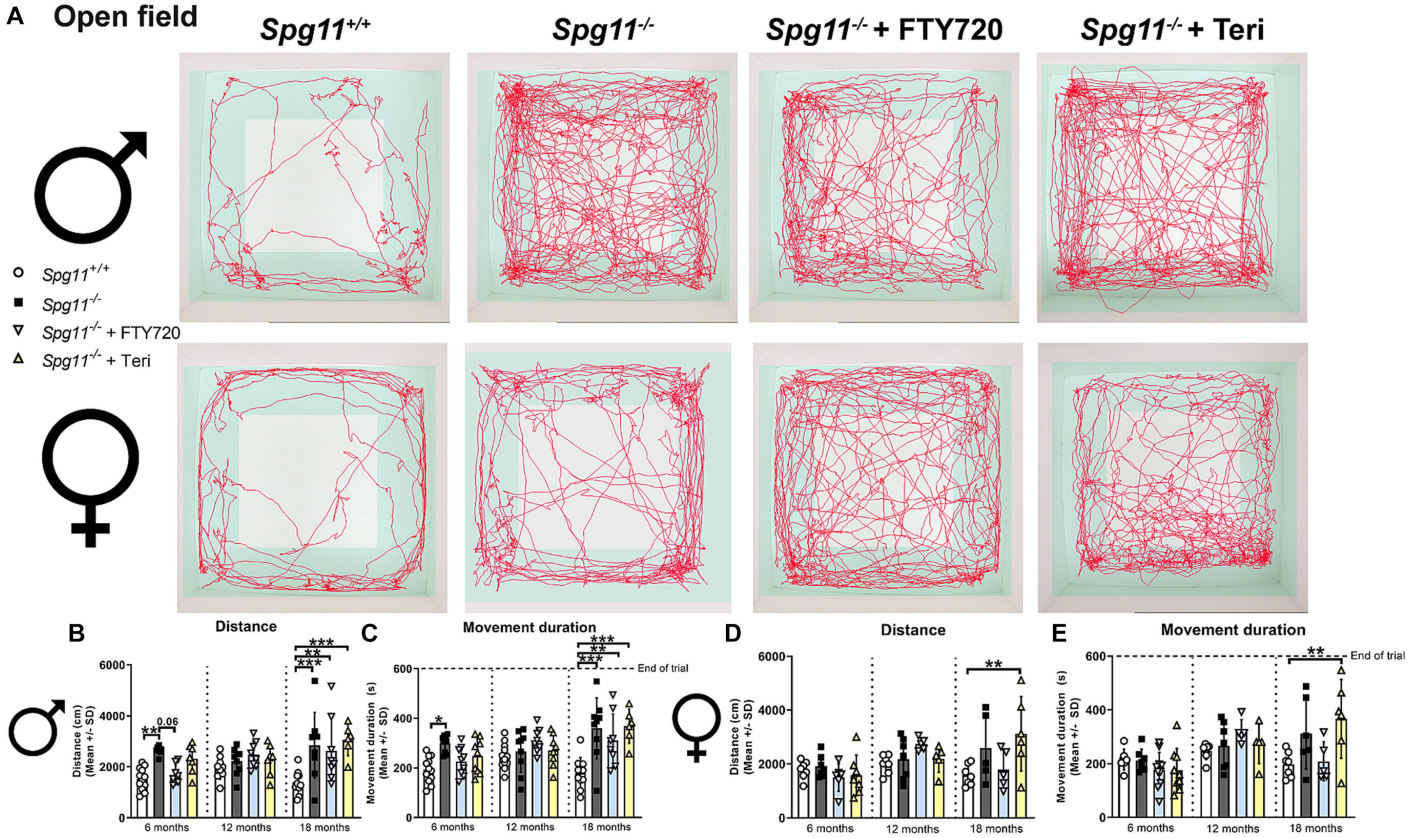
Treatment with fingolimod or teriflunomide does not reduce hyperactivity-like behavior of *Spg11^−/−^* mice. **(A)** Representative images of OF analysis of 18-month-old male (top row) and female (bottom row) *Spg11^+/+^*, *Spg11^−/−^,* and *Spg11^−/−^* mice treated with fingolimod (FTY720) or teriflunomide. Light square indicates the center zone of the arena, green area indicates the border zone. Red line represents the walking track. **(B)** While treatment with fingolimod reduces the walking distance and **(C)** movement duration of male *Spg11^−/−^* mice at 6 months neither treatment reduces them at 18 months [distance: *F*(11, 90) = 5.215, *p* < 0.0001; movement duration: *F*(11, 98) = 6.190, *p* < 0.0001]. **(D)** Treatment with fingolimod, but not teriflunomide, leads to a trend towards a reduced walking distance and **(E)** movement duration of 18-month-old female *Spg11^−/−^* mice [distance: *F*(11, 62) = 3.047, *p* = 0.0026; movement duration: *F*(11, 69) = 4.110, *p* = 0.0001]. Error bars represent standard deviations (circles, squares, triangles = value of one mouse). Significance of treated *Spg11^−/−^* mice compared to *Spg11^+/+^* and *Spg11^−/−^* mice is determined by one-way ANOVA and Sidak’s *post hoc* test (**p < 0.01, ***p < 0.001). Corresponding data from *Spg11^+/+^* and *Spg11^−/−^* mice, as shown in Figure 3, are presented here again.

**Figure 9:**
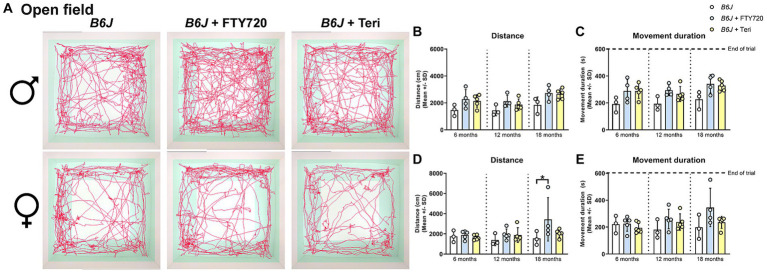
Treatment with fingolimod or teriflunomide increases aspects of hyperactivity-like behavior of *B6J* mice. **(A)** Representative images of OF analysis of 18-month-old male (top row) and female (bottom row) *B6J* and *B6J* mice treated with fingolimod (FTY720) or teriflunomide. Light square indicates the center zone of the arena, green area indicates the border zone. Red line represents the walking track. **(B)** Treatment with either immunomodulator leads to a tendency towards an increased walking distance and **(C)** movement duration of 18-month-old male *B6J* mice [distance: *F*(8, 27) = 3.246, *p* = 0.0102; movement duration: *F*(8, 27) = 3.073, *p* = 0.0135]. **(D)** Treatment with fingolimod, but not teriflunomide, increases the walking distance and **(E)** movement duration of 18-month-old female *B6J* mice [distance: *F*(8, 30) = 1.975, *p* = 0.0848; movement duration: *F*(8, 30) = 1.781, *p* = 0.1204]. Error bars represent standard deviations (circles = value of one mouse). Significance of treated *B6J* mice compared to *B6J* mice is determined by one-way ANOVA and Sidak’s *post hoc* test (*p < 0.05).

Using the OF analysis, we found that treatment with fingolimod, but not teriflunomide, reduced the walking distance and showed the same tendency for the movement duration, typically increased in male *Spg11^−/−^* mice, at 6 months of age (Figures 8A–C). No effect of treatment with either immunomodulator on these parameters was detectable at later ages (Figures 8A–C). Treatment of female *Spg11^−/−^* mice with fingolimod, but not teriflunomide, led to a trend towards a reduced walking distance and movement duration at 18 months (Figures 8A,D,E). While treatment with fingolimod did not change the exploratory behavior of male *B6J* mice, female *B6J* mice showed an increased walking distance and a tendency towards increased movement duration compared to untreated mice at 18 months (Figures 9A–E). Treatment with teriflunomide had no effect on the exploratory behavior of male or female *B6J* mice (Figures 9A–E).

**Figure 10:**
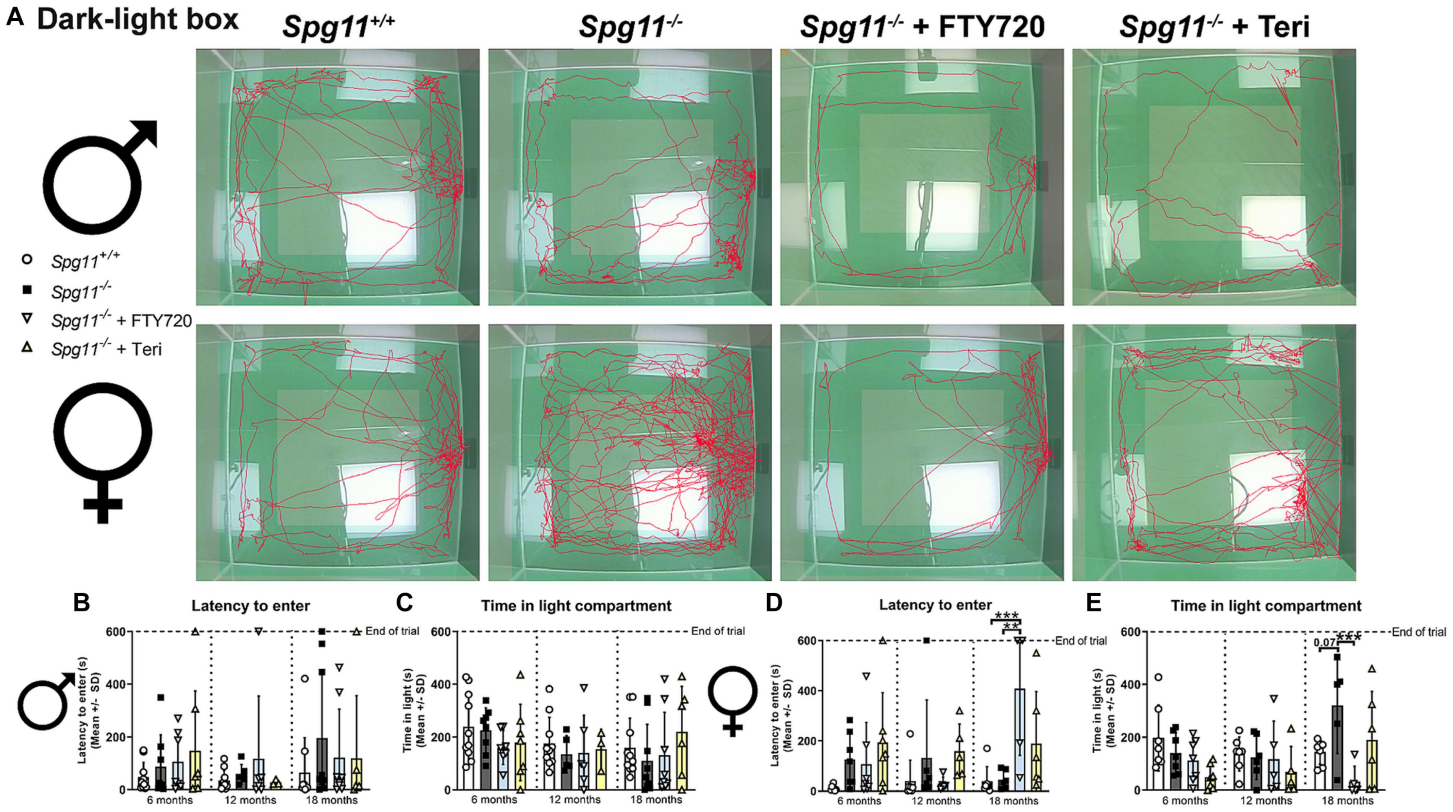
Treatment with fingolimod or teriflunomide restores anxiety-like behavior, typically reduced in female *Spg11^−/−^* mice. **(A)** Representative images of DLB analysis of 18-month-old male (top row) and female (bottom row) *Spg11^+/+^*, *Spg11^−/−^,* and *Spg11^−/−^* mice treated with fingolimod (FTY720) or teriflunomide. Light square indicates the center zone of the arena. Red line represents the walking track. **(B)** Treatment does not alter latency to enter, or **(C)** time spend in the light compartment of male *Spg11^−/−^* mice [latency to enter: *F*(11, 78) = 0.7479, *p* = 0.6896; time spend in light compartment: *F*(8, 30) = 1.781, *p* = 0.1204]. **(D)** Treatment with fingolimod, but not teriflunomide, increases the latency to enter of female *Spg11^−/−^* mice compared to wt and untreated knockout mice at 18 months [*F*(11, 62) = 2.880, *p* = 0.0041]. **(E)** Treatment with fingolimod reduces the time spend in the light compartment of 18-month-old female *Spg11^−/−^* mice, while teriflunomide shows the same tendency. Error bars represent standard deviations (circles, squares, triangles = value of one mouse). Significance of treated *Spg11^−/−^* mice compared to *Spg11^+/+^* and *Spg11^−/−^* mice is determined by one-way ANOVA and Sidak’s *post hoc* test (**p < 0.01, ***p < 0.001). Corresponding data from *Spg11^+/+^* and *Spg11^−/−^* mice, as shown in Figure 4, are presented here again.

**Figure 11:**
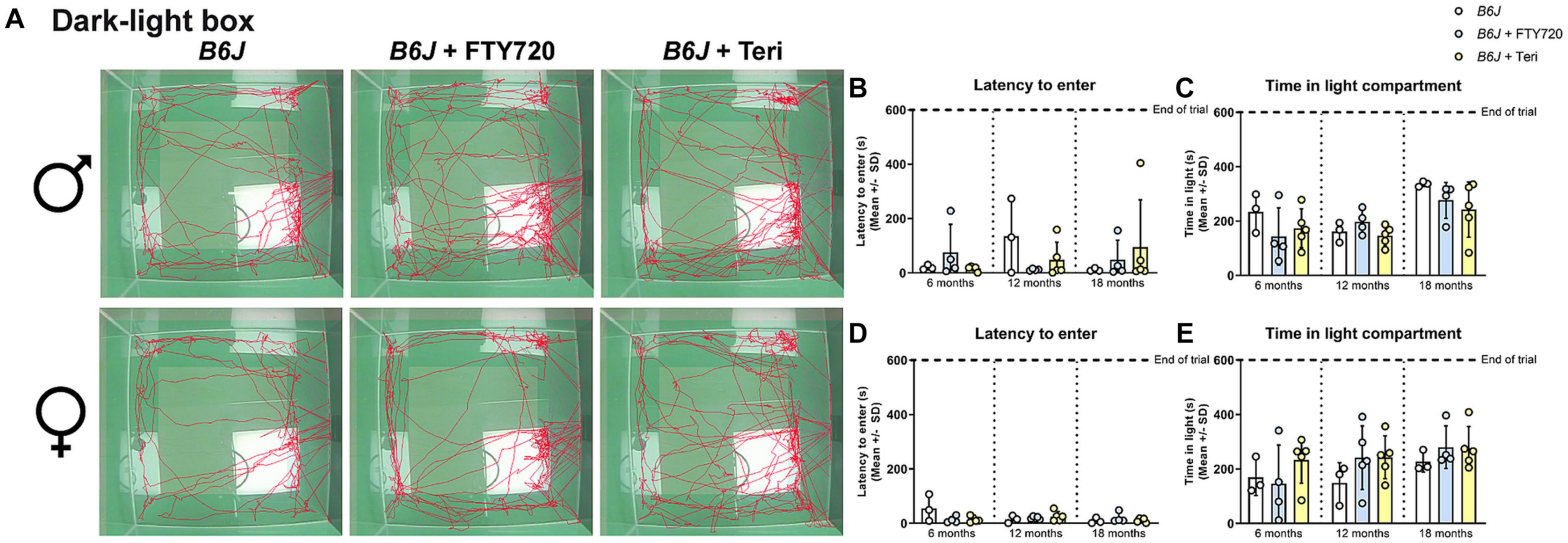
Treatment with fingolimod or teriflunomide does not alter anxiety-like behavior of *B6J* mice. **(A)** Representative images of DLB analysis of 18-month-old male (top row) and female (bottom row) *B6J* mice. **(B,C)** Treatment does not alter the latency to enter, or **(D,E)** time spend in the light compartment of male or female *B6J* mice [male: latency to enter: *F*(8, 27) = 0.8045, *p* = 0.6043; time spend in light compartment: *F*(8, 27) = 3.204, *p* = 0.0109; female: latency to enter: *F*(8, 28) = 1.926, *p* = 0.0955; time spend in light compartment: *F*(8, 28) = 1.193, *p* = 0.3386]. Error bars represent standard deviations (circles = value of one mouse). Significance of treated *B6J* mice compared to untreated *B6J* mice is determined by one-way ANOVA and Sidak’s *post hoc* test.

Regarding the anxiety-like behavior, neither treatment changed the behavior of male *Spg11^−/−^* mice compared to wt mice in the DLB analysis (Figures 10A–C). In female knockout mice, which typically show reduced anxiety-like behavior under non-treatment conditions, treatment with fingolimod led to an increased time to enter and less time spent in the light compartment compared to untreated *Spg11^−/−^* mice (Figures 10A,D,E). Of note, treatment of *Spg11^−/−^* mice with fingolimod also increased these parameters compared to wt mice (Figures 10A,D,E). Treatment with teriflunomide showed the same tendencies, albeit not to the same degree as fingolimod (Figures 10A,D,E). Treatment of male or female *B6J* mice with fingolimod or teriflunomide did not alter the latency to enter or time spent in light compartment (Figures 11A–E).

**Figure 12:**
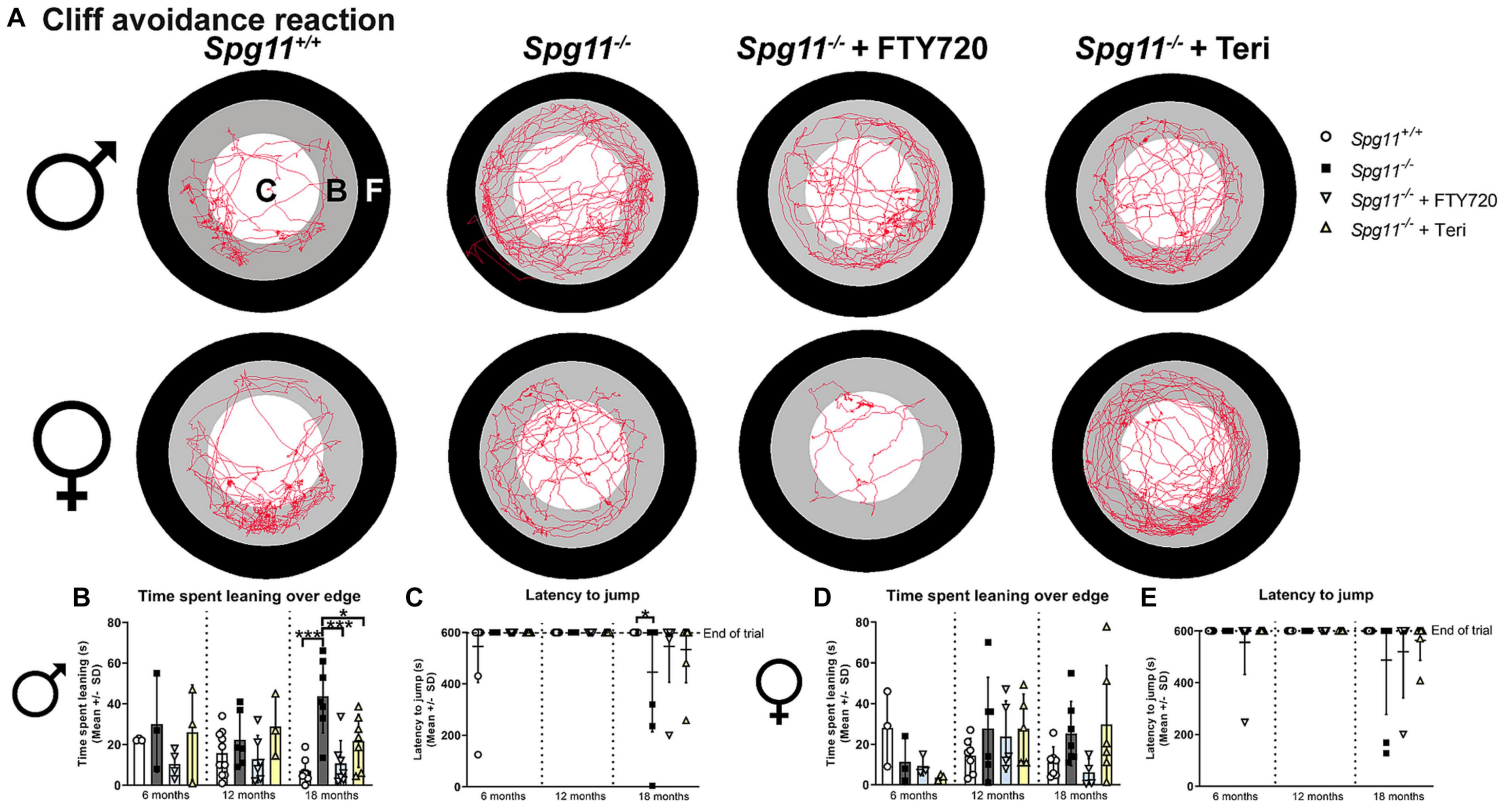
Treatment with fingolimod or teriflunomide reduces aspects of impulsivity-like behavior of *Spg11^−/−^* mice. **(A)** Representative images of CAR analysis of 18-month-old male (top row) and female (bottom row) *Spg11^+/+^*, *Spg11^−/−^* and *Spg11^−/−^* mice treated with fingolimod (FTY720) or teriflunomide. Red line represents the walking track (C = center zone, white; B = border zone, grey; F = floor zone, black). **(B)** Treatment reduces the time spend leaning over the edge of the platform, **(C)** but does not ameliorate the latency to jump off the platform of 18-month-old male *Spg11^−/−^* mice [time spend leaning: *F*(11, 55) = 3.931, *p* = 0.0003; latency to jump: *F*(11, 80) = 1.717, *p* = 0.0845] **(D)**. Treatment of female *Spg11^−/−^* mice with fingolimod, but not teriflunomide, shows a tendency towards a reduced time spend leaning over the edge of the platform at 18 months [*F*(11, 47) = 1.488, *p* = 0.1678]. **(E)** Treatment does not ameliorate the latency to jump off the platform of 18-month-old female *Spg11^−/−^* mice [*F*(11, 71) = 1.298, *p* = 0.2434]. Error bars represent standard deviations (circles, squares, triangles = value of one mouse). Significance of treated knockout mice compared to *Spg11^+/+^* and *Spg11^−/−^* mice is determined by one-way ANOVA and Sidak’s *post hoc* test (*p < 0.05, ***p < 0.001). Corresponding data from *Spg11^+/+^* and *Spg11^−/−^* mice, as shown in Figure 5, are presented here again.

**Figure 13:**
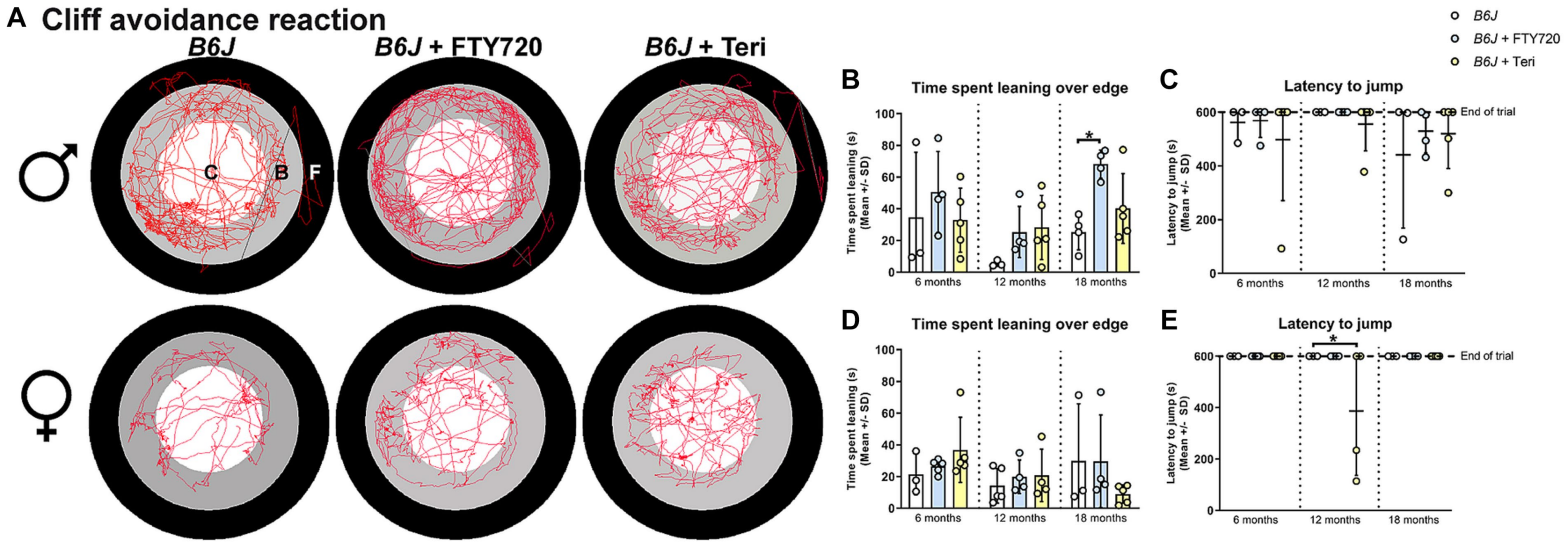
Treatment with fingolimod or teriflunomide increases aspects of impulsivity-like behavior of *B6J* mice. **(A)** Representative images of CAR analysis of 18-month-old male (top row) and female (bottom row) *B6J* and *B6J* mice treated with fingolimod (FTY720) or teriflunomide. Red line represents the walking track (C = center zone, white; B = border zone, grey; F = floor zone, black). **(B)** Treatment with fingolimod increases the time male *B6J* spend leaning over the edge of the platform compared to untreated mice at 18 months [*F*(8, 28) = 2.682, *p* = 0.0252]. **(C)** Untreated male *B6J* mice show jumping behavior and treatment does not alter this [*F*(8, 27) = 0.4806, *p* = 0.8591]. **(D)** Treatment does not alter the time female *B6J* mice spend leaning over the edge of the platform [*F*(8, 29) = 1.117, *p* = 0.3808]. **(E)** Treatment with teriflunomide, but not fingolimod, reduces the latency to jump off the platform of 12-month-old female *B6J* mice compared to untreated mice [*F*(8, 27) = 2.886, *p* = 0.0185]. Error bars represent standard deviations (circles = value of one mouse). Significance of treated *B6J* mice compared to untreated *B6J* mice is determined by one-way ANOVA and Sidak’s *post hoc* test (*p < 0.05).

Similar to our findings in *Rag1*-deficient mice, treatment with fingolimod or teriflunomide reduced the time male *Spg11^−/−^* mice spent leaning over the edge of the platform, while the latency to jump remained unchanged in the CAR analysis (Figures 12A–C). In female *Spg11^−/−^* mice, treatment with fingolimod, but not teriflunomide, led to a tendency towards a reduced time spent leaning over the edge of the platform, while the latency to jump remained unaffected by both treatments (Figures 12A,D,E). Conversely, treatment of male *B6J* mice with fingolimod increased the time spent leaning over the edge of the platform at 18 months (Figures 13A,B). Surprisingly, we detected a reduced latency to jump off the platform in untreated male *B6J* mice, that was not altered by treatment with either immunomodulator (Figure 13C). In female *B6J* mice, the time spent leaning over the edge of the platform remained unaffected, but treatment with teriflunomide led to a reduced latency to jump off the platform when compared to untreated mice at 12 months (Figures 13A,D,E). Treatment with fingolimod did not lead to behavioral changes of female *B6J* mice in the CAR analysis (Figures 13A,D,E).

In summary, we here show that pharmacological immunomodulation improves distinct behavioral abnormalities, especially related to social behavior, of *Spg11^−/−^* mice.

### Degeneration and preservation of Purkinje cells correlate with development and mitigation, respectively, of behavioral abnormalities of *Spg11^−/−^* mice

**Figure 14:**
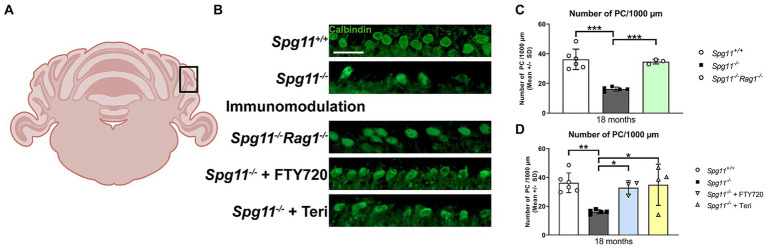
Genetic or pharmacological immunomodulation preserves Purkinje cell numbers of *Spg11^−/−^* mice. **(A)** Schematic representation of the mouse cerebellum. Black box indicates the area in which Purkinje cells (PC) were counted. Created with BioRender. **(B)** Representative images of Calbindin^+^ PC in the cerebellum of 18-month-old *Spg11^+/+^* (top row), *Spg11^−/−^*(2nd row), *Spg11*^−/−^*Rag1^−/−^*(3rd row) and *Spg11^−/−^* mice treated with fingolimod (FTY720) (4th row) or teriflunomide (bottom row). Scale bar: 50 μm. **(C)** *Spg11^−/−^* mice show loss of PC compared to wt littermates at 18 months and *Rag1*-deficiency [*F*(2, 11) = 27.52, *p* < 0.0001] or **(D)** treatment with fingolimod (FTY720) or teriflunomide preserves PC numbers [*F*(3, 14) = 6.759, *p* = 0.0048]. Error bars represent standard deviations (circles, squares, triangles = mean value of one mouse). Significance of *Spg11^−/−^Rag1^−/−^* and treated *Spg11^−/−^* mice compared to *Spg11^+/+^* and *Spg11^−/−^* mice are determined by one-way ANOVA and Sidak’s *post hoc* test (*p < 0.05, **p < 0.01, ***p < 0.001). (*Spg11^+/+^* mice = 6*; Spg11^−/−^* mice = 5; *Spg11^−/−^Rag1^−/−^* mice = 3; *Spg11^−/−^* mice+ FTY720 = 3; *Spg11^−/−^* mice + Teri = 4). Corresponding data from *Spg11^+/+^* and *Spg11^−/−^* mice are presented in **(C,D)**.

Correct function of cerebellar Purkinje cells (PC) is implicated in social behavior of humans and mice (Bauman and Kemper, 2005; Amaral et al., 2008; Tsai et al., 2012; Reith et al., 2013; Cutando et al., 2022). It has been previously demonstrated that *Spg11*^−/−^ mice show loss of PC (Branchu et al., 2017), and we detected high levels of inflammation in the cerebellum (Hörner et al., 2022). Therefore, we wanted to investigate whether genetic inactivation of the adaptive immune system affects PC loss, possibly explaining the ameliorated social behavior of *Spg11*^−/−^*Rag1*^−/−^ mice. Agreeing with the previously reported findings, we saw a pronounced loss of PC in *Spg11*^−/−^ mice compared to wt littermates at 18 months (Figures 14A–C), and indeed, *Rag1*-deficieny prevented this loss (Figures 14B,C). Importantly, treatment with fingolimod or teriflunomide also significantly attenuated the loss of PC in the cerebellum of *Spg11^−/−^* mice (Figures 14B,D).

In summary, we here show that genetic or pharmacological immunomodulation preserves PC numbers in the cerebellum of *Spg11^−/−^* mice.

## Discussion

We previously showed that secondary inflammation by the adaptive immune system contributes to disease progression in a mouse model of SPG11 (Hörner et al., 2022). This comprised histopathological changes and distinct gait abnormalities related to coordination, partially reflecting abnormal gait parameters seen in patients (Hörner et al., 2022). Here, we further extend our analysis by shifting the research focus to the impact of secondary inflammation on behavioral abnormalities related to neuropsychological features typically detected in SPG11 patients.

### *Spg11^−/−^* mice display distinct behavioral alterations, partially reflecting neuropsychological features of patients

In humans, neuropsychological abnormalities are accompanying symptoms in many CNS diseases like AD, PD, MS, multiple system atrophy and HSPs (Becker et al., 1988; St Clair et al., 1988; Brassington and Marsh, 1998; Osmolak et al., 2012; Barcelos et al., 2018; Faber et al., 2018). However, behavioral involvement remains an insufficiently researched area and the underlying pathomechanisms remain mainly elusive. Here, we established a behavioral test battery that revealed abnormalities in social, hyperactivity-like, anxiety-like, and impulsivity-like behavior of *Spg11*^−/−^ mice, all being relevant for SPG11 patients (Wijemanne et al., 2015; Faber et al., 2018; Utz et al., 2022; Klebe et al., unpublished data). Generally, we noted that most behavioral abnormalities were detected at higher ages (e.g., from month 12 or 18 onwards), faithfully reflecting the relatively later onset of many behavioral features in patients and confirming the suitability of the test battery selected.

Learning and memory difficulties are a prominent feature of SPG11 patients (Faber et al., 2018; Utz et al., 2022) and previously published work provided evidence that *Spg11^−/−^* mice show cognitive deficits (Branchu et al., 2017). We could not detect defects in memory and recognition of *Spg11^−/−^* mice. However, the Y-maze test used by Branchu et al. relied on spatial memory, while the here used NOR investigates recognition without a spatial memory component. It is therefore possible, that the two forms of memory acquisition are affected differently in this mouse model. Furthermore, *Spg11^−/−^* mice showed signs of hyperactivity-like behavior and previous studies could provide evidence that SPG11 patients show signs of attention deficit/hyperactivity syndrome (ADHD) (Wijemanne et al., 2015). Interestingly, female *Spg11^−/−^* mice showed reduced anxiety-like behavior, that was not detectable in male knockout mice, while male *Spg11^−/−^* mice showed more pronounced impulsivity-like behavior. While the exact mechanisms responsible for the sex differences in behavioral changes of *Spg11*^−/−^ mice remain to be identified, previous studies offered multiple putative explanations for sex differences in neuropsychological disease manifestations, including CNS-associated immune reactions (Osborne et al., 2018; Bangasser and Cuarenta, 2021). Regarding the here identified altered features of social behavior, it is tempting to speculate that impaired olfactory function in the mutants might be a possible reason, as impaired olfaction is an established symptom of other neurodegenerative disorders, like AD (Murphy, 2019). Further studies in patients and the corresponding mouse model are required to address this question.

In summary, our SPG11 mouse model reflects many aspects of mostly later-onset neuropsychological features detected in SPG11 patients.

### Immunomodulation improves distinct behavioral abnormalities of *Spg11^−/−^* mice

We found that *Rag1*-deficiency and treatment with the immunomodulators fingolimod (FTY720) or teriflunomide robustly attenuated the abnormal social behavior of male and female *Spg11^−/−^* mice. A study by McGowan et al. (2011) indicated that genuine *Rag1*-deficient mice show an impaired social recognition memory. Importantly, these experiments focused largely on social memory, and social behavior of *Rag1*-deficient mice was comparable to those of wt mice after a retention phase of 30 min (McGowan et al., 2011), indicating that *Rag1*-deficiency does not generally alter social behavior. Of note, especially treatment with fingolimod has been shown to be beneficial in other disease models regarding social abnormalities, e.g., it attenuated social deficits in a rat model of autism spectrum disorder (Wu et al., 2017), and in mouse models of experimental autoimmune encephalomyelitis (EAE) (Baert et al., 2019), and systemic lupus erythematosus (de Bruin et al., 2016; Shi et al., 2018).

Regarding anxiety-related parameters, genetic and immunomodulatory approaches counteracted the reduced anxiety-like behavior, typically detected in female *Spg11^−/−^* mice. Some studies indicate that *Rag1*-deficiency leads to increased anxiety- and depression-like behavior in mice (Rattazzi et al., 2013; Smith et al., 2014), and indeed *Rag1*-deficiency increased anxiety-like behavior of wt mice in our study. Conversely, fingolimod treatment has been shown to reduce anxiety-like behavior in mouse EAE (Bonfiglio et al., 2017), while other studies provided evidence that treatment with fingolimod did not affect anxiety-like behavior in other mouse or rat models (di Nuzzo et al., 2015; Leo et al., 2017). In line with these findings, we did not detect an effect of treatment with immunomodulators on anxiety-like behavior of *B6J* mice, although we acknowledge the low number of mice as a possible limitation of this result. It has to be noted that there is evidence of a mechanistic link between anxiety-like behavior and microglial activation, especially in the amygdala and the hippocampus (Sawada et al., 2014; Stein et al., 2017; Wang et al., 2018). However, we did not analyze microglia in these compartments. It should be subject of future experiments to determine the connection of *Rag1*-deficiency/pharmacological immune modulation, microglial activation, and anxiety-like behavior.

Regarding impulsivity-like behavior, *Rag1*-deficiency and pharmacological treatment could reduce the time *Spg11^−/−^* mice spent leaning over the edge of the platform. Indeed, it has been shown that inflammatory markers are elevated in humans and mice that display traits linked to impulsivity and aggression (Beurel and Jope, 2014; Logsdon et al., 2016; Kim et al., 2020), and reducing neuroinflammation in a rat model of traumatic brain injury led to a significant reduction of impulsivity-like behavior in the elevated platform maze test (Logsdon et al., 2016).

The role of T-lymphocytes as pathogenic amplifiers in genetically mediated diseases has been well-documented (Groh et al., 2013, 2016, 2017, 2018, 2023; Groh and Martini, 2017; Hörner et al., 2022; Abdelwahab et al., 2023). Additionally, research has implicated microglia and their activation states in disease progression, particularly in neurodegenerative and neurodevelopmental disorders (Lukens and Eyo, 2022; Matuleviciute et al., 2023). Notably, prior investigations of the *Spg11^−/−^* mouse model revealed elevated numbers and activation of microglia, suggesting a potential contribution to behavioral abnormalities (Hörner et al., 2022). While the present study focused on the impact of targetable adaptive immune cells, their interaction with microglia should be explored in future experiments.

Taken together, the here presented data provide evidence that behavioral abnormalities in this mouse model, especially related to social behavior, can indeed be mediated, or aggravated by secondary inflammation.

### Preservation of Purkinje cells by immune modulation correlates with ameliorated social behavior of *Spg11^−/−^* mice

Structural abnormalities or loss of PC in mice or humans are often associated with altered social behavior (Bauman and Kemper, 2005; Amaral et al., 2008; Tsai et al., 2012; Reith et al., 2013; Sudarov, 2013; Cutando et al., 2022). The here presented mouse model shows axonal damage in the cerebellum (Hörner et al., 2022) and loss of PC (Branchu et al., 2017). According to our previous (Hörner et al., 2022) and present study, these degenerative changes are mediated by disease-amplifying inflammation most likely related to infiltrating CD8^+^ T-lymphocytes. Thus, mitigation of behavioral changes by immune modulation may be related to dampening of immune-driven damage and loss of PC.

What is the link between SPG11-deficiency and immune-mediated loss of PC causing behavioral changes? It has been previously described that mouse mutants for myelin genes also develop secondary, disease-amplifying inflammation (Ip et al., 2006, 2012; Kroner et al., 2010; Groh et al., 2016; Janova et al., 2018). Similar observations have been made in aging white matter (Groh et al., 2021b) and perturbed myelin has been identified as a risk for axonal degeneration, neuron loss and behavioral decline, implicating inflammation (Schaffner et al., 2023; Groh et al., 2023). Based on the finding that spatacsin is also robustly expressed by oligodendrocytes (Sjostedt et al., 2020), it is plausible to assume that mutant SPG11 or SPG11-deficiency might lead to molecularly altered myelin sheaths in the CNS. Indeed, we previously observed myelin abnormalities in the SPG11 mouse model, in the form of redundant and fragmented myelin profiles, possibly indicative of an altered molecular composition (Hörner et al., 2022). Molecularly altered myelin may be targeted by CD8^+^ T-lymphocytes, leading to axonal damage and subsequent neuronal loss in case of the PC. Alternatively, or additionally, it is possible that neurons are the primary target of inflammation, as spatacsin is strongly expressed by these cells (Murmu et al., 2011; Perez-Branguli et al., 2014; Pozner et al., 2020). This view is in line with our observation that lymphocytes invade not only white, but also grey matter compartments of SPG11 mutants (Hörner et al., 2022).

Taken together, our findings support the notion that inflammation-mediated cerebellar dysfunction plays a prominent role in SPG11, not only regarding gait abnormalities (Hörner et al., 2022), but also in the development of altered social behavior. Additionally, this study further strengthens the hypothesis that inflammation by CD8^+^ T-lymphocytes, leading to axonal perturbation and consequent loss of neuronal cell bodies, has an overarching role in the disease progression of many CNS related diseases (Groh et al., 2017; Hörner et al., 2022; Abdelwahab et al., 2023), disorders presenting with neuropsychological features, and aging (Groh et al., 2021b). Nevertheless, future studies should carefully investigate the long-term effects (both putative beneficial and adverse effects) of treatment with immunomodulators in SPG11.

## Conclusion

We previously demonstrated that neuroinflammation is a targetable amplifier of neuropathological changes and gait performance in a mouse model for SPG11. As neuropsychological features are also strongly limiting quality of life for both SPG11 patients and their relatives, we here focused on these clinically highly relevant symptoms. We found that some behavioral abnormalities of *Spg11^−/−^* mice are indeed mediated or amplified by neuroinflammation and can be mitigated by genetic targeting of lymphocytes and, more importantly, clinically approved immunomodulators. These findings may be of high translational relevance as they could pave the way for using immunomodulators as a treatment option for improving movement and neuropsychological aspects in SPG11.

## Data availability statement

The original contributions presented in the study are included in the article/Supplementary material, further inquiries can be directed to the corresponding authors.

## Ethics statement

The animal study was approved by Government of Lower Franconia, Germany. The study was conducted in accordance with the local legislation and institutional requirements.

## Author contributions

MH: Conceptualization, Methodology, Writing – review & editing, Writing – original draft, Data curation, Formal analysis, Investigation, Project administration, Visualization. SP: Formal analysis, Investigation, Methodology, Writing – review & editing. JB: Methodology, Writing – review & editing, Resources. GS: Methodology, Resources, Writing – review & editing. FD: Methodology, Resources, Writing – review & editing. SK: Conceptualization, Methodology, Writing – review & editing. JG: Conceptualization, Funding acquisition, Methodology, Supervision, Writing – review & editing. RM: Conceptualization, Funding acquisition, Methodology, Project administration, Supervision, Writing – original draft, Writing – review & editing.

## Footnotes

^1^https://www.randomizer.org/, last accessed 2023/04/29.
