# Supplementary material for: Clinically approved immunomodulators ameliorate behavioral changes in a mouse model of hereditary spastic paraplegia type 11
Source: Front Neurosci. 2024 Feb 16;18:1299554. doi: 10.3389/fnins.2024.1299554 (PMC10904495; doi:10.3389/fnins.2024.1299554)
Supplement: Supplementary file 1 [file Data_Sheet_1.docx]

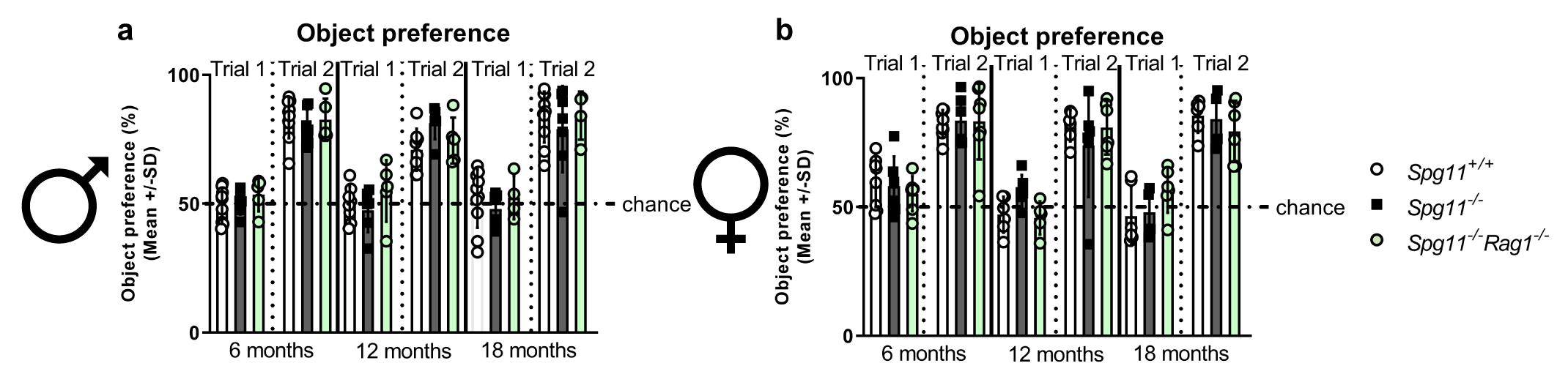
**Supplementary Figure 1: *Spg11^-/-^* or *Spg11^-/-^Rag1^-/-^* mice do not show altered object preference in the NOR analysis.** **(a)** Object preference of male or **(b)** female *Spg11^-/-^* and *Spg11^-/-^Rag1^-/-^* mice is not altered in NOR compared to wt mice (male: *F* (17, 106) = 20.91, *P* < 0.0001; female: *F* (17, 91) = 13.49, *P* < 0.0001). Chance indicates equal time spent with both objects (50%). Error bars represent standard deviations (circles, squares = value of one mouse). Significance of *Spg11^-/-^* compared to *Spg11^+/+^* and *Spg11^-/-^Rag1^-/-^* compared to *Spg11^+/+^* and *Spg11^-/-^* mice is determined by one-way ANOVA and Sidak’s post hoc test.


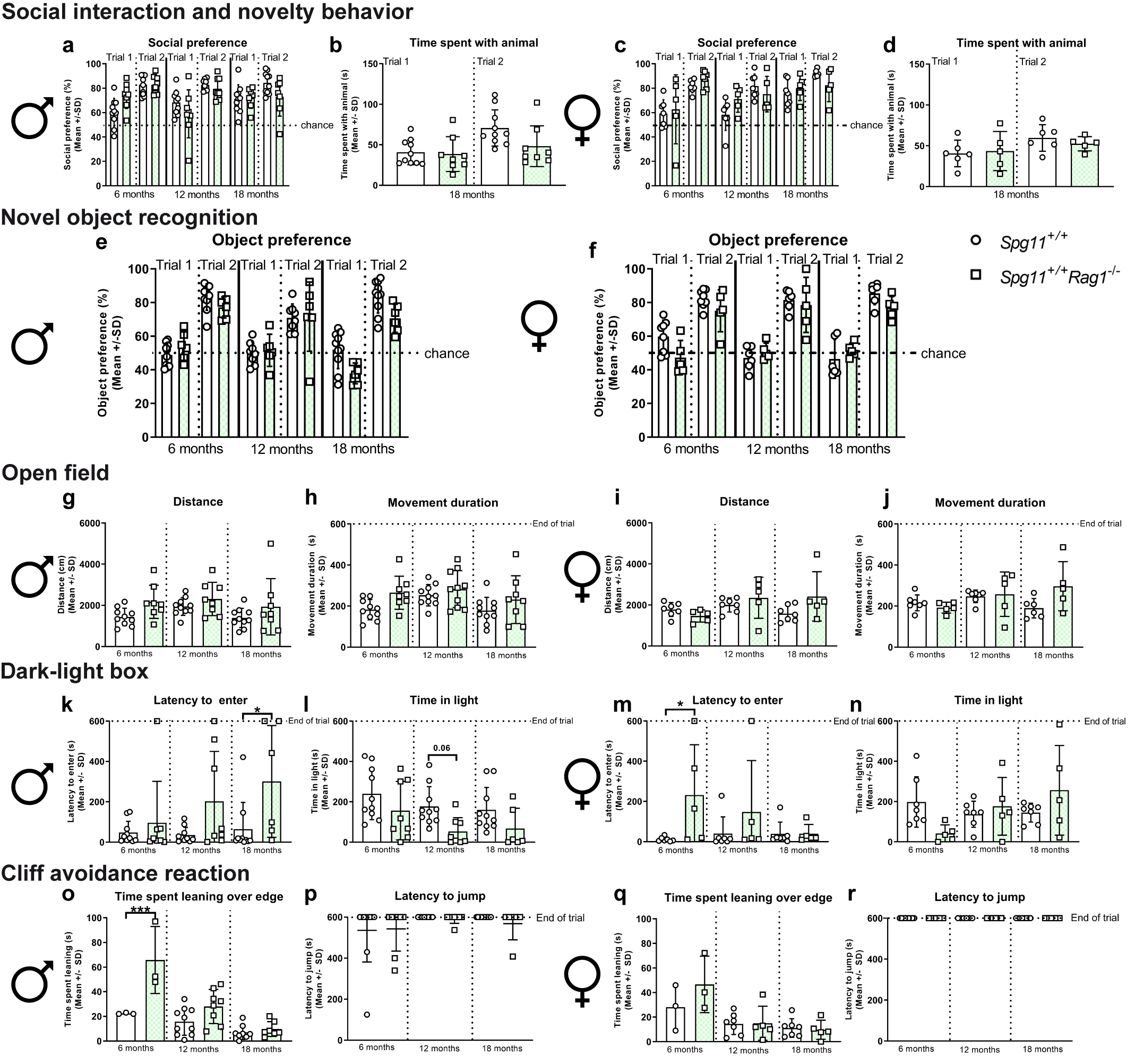

**Supplementary Figure 2: *Rag1-*deficiency increases anxiety-like behavior of *Spg11^+/+^* mice while other parameters remain unaffected. (a, b)** *Rag1-*deficiency does not alter social interaction or novelty behavior of male or **(c, d)** female *Spg11^+/+^* mice (male: social preference: *F* (11, 91) = 5.141, *P* < 0.0001; time spent with animal: *F* (3,32) = 4.620, P = 0.0085; female: social preference: *F* (11, 61) = 5.049, *P* < 0.0001; time spent with animal: *F* (3, 18) = 1.391, P = 0.2508). **(e)** *Rag1-*deficiency does not alter object preference in NOR of male or **(f)** female *Spg11^+/+^* mice (male: *F* (11, 80) = 19.94, *P* < 0.0001; female: *F* (11, 52) = 15.74, *P* < 0.0001). (**g, h)** *Rag1-*deficiency does not alter distance traveled or movement duration of male or **(i, j)** female *Spg11^+/+^* mice (male: distance: *F* (5, 48) = 2.230, *P* = 0.0664; movement duration: *F* (5, 50) = 3.164, *P* = 0.0147; female: distance: *F* (5, 30) = 2.021, *P* = 0.1042; movement duration: *F* (5, 29) = 0.2.041, *P* =0.1022). **(k)** *Rag1-*deficiency increases the latency to enter **(l)** decreases the time spent in the light compartment of 18-month-old male *Spg11^+/+^* mice at 12 months, while showing the same tendency at 18 months (latency to enter: *F* (5, 46) = 2.840, *P* = 0.0257; time spend in light compartment: *F* (5, 46) = 3.294, *P* = 0.0126). (**m)** *Rag1-*deficiency increases the latency to enter of 6-month-old female *Spg11^+/+^* mice and **(n)** shows a tendency towards a decreased time spent in the light compartment at the same age (latency to enter: *F* (5, 30) = 2.069, *P* = 0.0972; time spend in light compartment: *F* (5, 31) = 1.581, *P* = 0.1946). **(o)** *Rag1-*deficiency increases the time spent leaning over the edge of the platform of 6-month-old male *Spg11^+/+^* mice, while this parameter is unaffected at all other ages (*F* (5, 33) = 13.66, *P* < 0.0001). **(p)** *Rag1-*deficiency does not alter the latency to jump off the platform of male (*F* (5, 44) = 1.011, *P*= 0.4229), and **(q, r)** the time spent leaning over the edge of the platform or the latency to jump off the platform of female *Spg11^+/+^* mice (time spent leaning: *F* (5, 23) = 4.106, *P*= 0.082; latency to enter: samples all have a standard error of zero). Chance indicates equal time spent with both objects (50%). Error bars represent standard deviations (circles, squares, triangles = value of one mouse). Significance of *Spg11^+/+^Rag1^-/-^* mice compared to *Spg11^+/+^* mice is determined by one-way ANOVA and Sidak’s post hoc test (**p* < 0.05, ****p* < 0.001). Corresponding data from *Spg11^+/+^* mice as shown in figures 2, 3, 4, 5, and supplementary figure 1, are presented here again.

**
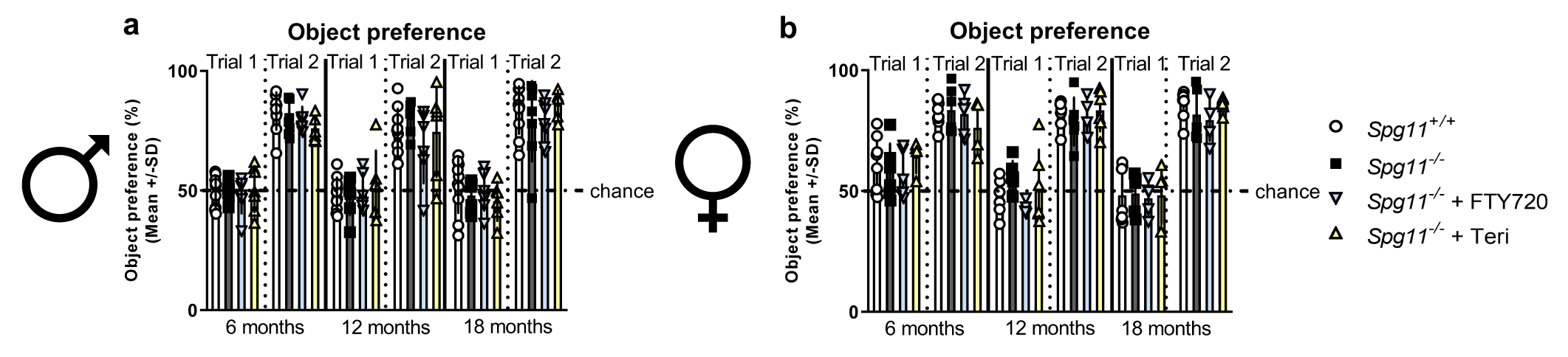
Supplementary Figure 3: Treatment with fingolimod or teriflunomide does not alter object preference in the NOR analysis of *Spg11-/-* mice. (a)** Treatment with neither fingolimod (FTY720) nor teriflunomide impacts the object preference in NOR of male or **(b)** female *Spg11^-/-^* mice (male: *F* (23, 158) = 20.41, *P* < 0.0001; female: *F* (23, 118) = 17.83, *P* < 0.0001). Chance indicates equal time spent with both objects (50%). Error bars represent standard deviations (circles, squares, triangles = value of one mouse). Significance of treated knockout mice compared to *Spg11^+/+^* and untreated *Spg11^-/-^* mice is determined by one-way ANOVA and Sidak’s post hoc test. Corresponding data from *Spg11^+/+^* and *Spg11^-/-^* mice as shown supplementary figure 1, are presented here again.

**
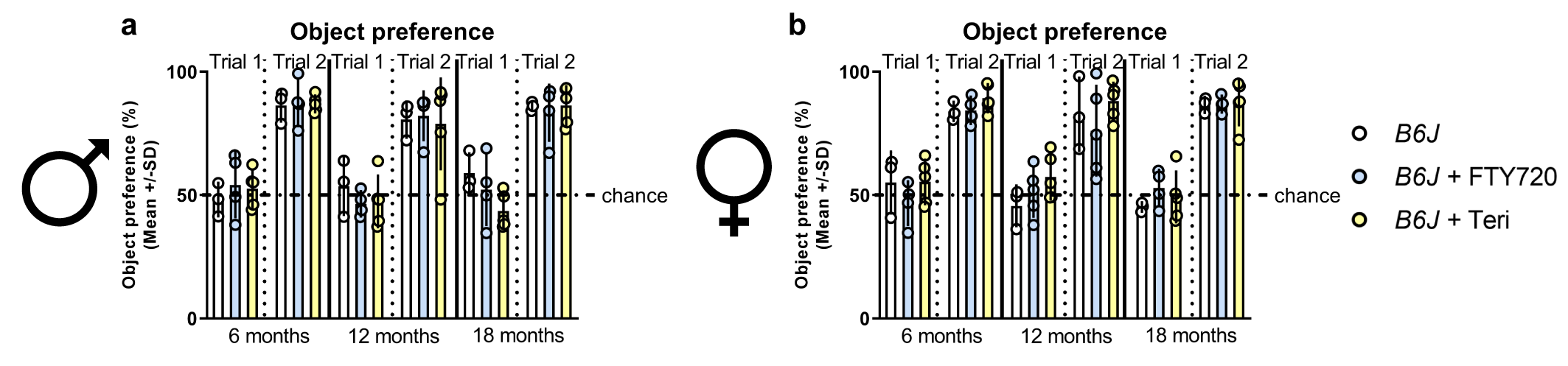
Supplementary Figure 4: Treatment with fingolimod or teriflunomide does not alter object preference in the NOR analysis of *B6J* mice. (a)** Early treatment with neither fingolimod (FTY720) nor teriflunomide impacts the object preference in NOR of male **(b)** or female *B6J* mice (male: *F* (17, 54) = 12.98, *P* < 0.0001; female: *F* (17, 56) = 14.87, *P* < 0.0001). Chance indicates equal time spent with both objects (50%). Error bars represent standard deviations (circles = value of one mouse). Significance of treated *B6J* mice compared to *B6J* mice is determined by one-way ANOVA and Tukey’s post hoc test.
